# Chemical Constituents from *Scindapsus officinalis* (Roxb.) Schott. and Their Anti–Inflammatory Activities

**DOI:** 10.3390/molecules23102577

**Published:** 2018-10-09

**Authors:** Hongjing Dong, Yanling Geng, Xueyong Wang, Xiangyun Song, Xiao Wang, Jinqian Yu

**Affiliations:** 1 Shandong Key Laboratory of TCM Quality Control Technology, Shandong Analysis and Test Center, Qilu University of Technology (Shandong Academy of Sciences), Jinan 250014, China; donghongjing_2006@163.com (H.D.); gengyanling@163.com (Y.G.); sxyun198806@163.com (X.S.); wangx@sdas.org (X.W.); 2 College of Chinese Mareria Medica, Beijing University of Chinese Medicine, Beijing 100102, China; xueyongwang@bucm.edu.cn

**Keywords:** *Scindapsus officinalis*, monoterpene glycoside, phenyl glycoside, caffeoyl derivative, anti-inflammatory activity

## Abstract

One new monoterpene glycoside (**1**), one new phenyl glycoside (**2**), one new caffeoyl derivative (**3**), were isolated from *Scindapsus officinalis* (Roxb.) Schott., along with four known compounds (**4–7**). Structures of the isolated compounds were elucidated by extensive analysis of spectroscopic data, especially 2D NMR data and comparison with literatures. All isolates were evaluated for anti-inflammatory activity against nitric oxide (NO) production in vitro. Compounds **3** and **7** exhibited moderate inhibitory effects on NO production with IC_50_ values of 12.2 ± 0.8 and 18.9 ± 0.3 μM, respectively.

## 1. Introduction

**Figure 1:**
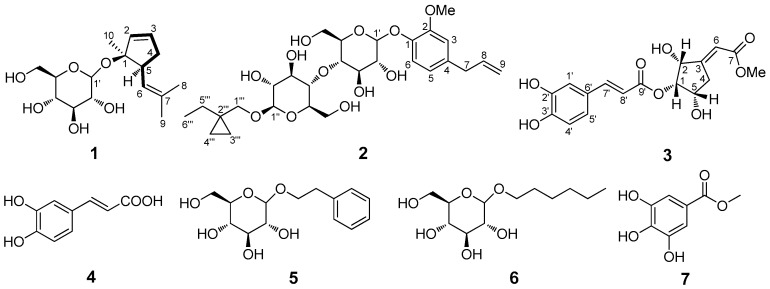
Structures of compounds **1**–**7** and ^1^H-^1^H COSY (bold) of **1**.

The family of Araceae plants, comprising 115 genera and more than 2100 species, is distributed all over the world. However, 90% of the species is predominantly found in tropical America, India, Malaya, and South China [1]. Araceae plants are generally recognized as a rich source of biologically active and structurally unique compounds, such as alkaloids, phenols, saponins, sterols and so on [2,3,4,5,6]. The chemical diversities reported for Araceae plants are fascinating from a pharmacological viewpoint. *Scindapsus officinalis* (Roxb.) Schott. is a large, epiphytic, and perennial liana in the family Araceae, which usually climbs up to trees and rocks using aerial roots and is mainly distributed in the south-central regions of China and the tropical parts of India. The whole plant of *S. officinalis* has been used in Guizhou, Yunnan and Guangxi Provinces in China as ethnomedicine to treat bone fractures, arthralgia, bronchitis, and pertussis [7]. According to its usage in ethnomedicine, the whole plant of *S. officinalis* is confirmed to show anti-inflammatory activity. However, a literature search indicates that limited studies have been conducted on the anti-inflammatory chemical constituents of the whole plant up to now, including only several chromone derivatives and alkaloids obtained by our group [8,9]. As part of our ongoing commitment to search for novel, structurally intriguing, and anti-inflammatory natural products from *S. officinalis*, one new monoterpene glycoside (**1**), one new phenyl glycoside (**2**), one new caffeoyl derivative (**3**), and four known compounds (**4**–**7**) (Figure 1), were isolated. Herein, the isolation and structural elucidation toward the new compounds, as well as the inhibitory effect of all the isolated compounds on lipoplysaccharide (LPS)-induced NO production in RAW 264.7 cells, are described.

## 2. Results

The condensed 95% ethanol (EtOH) extract of *S*. *officinalis* was suspended in 20% ethanol solution and successively subjected to multi-solvent partition with petroleum ether, ethyl acetate (EtOAc), and *n*-butanol (*n*-BuOH). The EtOAc fraction was subjected to diverse column chromatography to furnish seven compounds, including one new monoterpene glycoside (**1**), one new phenyl glycoside (**2**), one new caffeoyl derivative (**3**), and four known compounds (**4**–**7**). By comparison with previous NMR data in the literature, the known compounds were identified as caffeic acid (**4**) [10], 2-phenylethyl *β*-d-Glucopyranoside (**5**) [11], hexyl-*O*-*β*-d-Glucopyranoside (**6**) [12], and methyl gallate (**7**) [13], which were isolated from *S. officinalis* for the first time.

Compound **1** was obtained as a brown gum. Its molecular formula was established as C_16_H_26_O_6_ from its ^13^C-NMR data and a sodium adduct molecular ion peak at *m*/*z* 337.1657 [M + Na]^+^ in the HRESIMS (positive ion mode) spectrum (calcd for C_16_H_26_NaO_6_, 337.1622), representing four degrees of unsaturation. The ^1^H-NMR spectrum of **1** (Table 1) indicated the presence of three olefinic protons at *δ*_H_ 5.98 (1H, dd, *J* = 11.2, 17.6 Hz, H-2) and 5.09 (2H, overlapped, H-3, 6), and three methyls at *δ*_H_ 1.61 (3H, s, Me-8), 1.54 (3H, s, Me-9) and 1.22 (3H, s, Me-10). In addition, one obvious anomeric proton at *δ*_H_ 4.13 (1H, d, *J* = 8.0 Hz, H-1′) which correlated with carbon signal at *δ*_C_ 105.1 (C-1′) observed in the HSQC spectrum, indicated one pyranose moiety. The relatively large coupling constant suggested that the pyranose moiety possessed a *β*-glycosidic bond. The ^13^C-NMR data of **1** exhibited a total of sixteen carbon signals (Table 1) assignable via HSQC spectrum to three methyls, two methylenes (including one oxygenated carbon at *δ*_C_ 61.7 (C-6′)), nine methines [including three olefinic carbons at *δ*_C_ 144.1 (C-2), 114.1 (C-3), 125.3 (C-6), five oxygenated methines, and one anomeric carbon at *δ*_C_ 98.2 (C-1′)], and two quaternary carbons (including one olefinic carbon at *δ*_C_ 130.8 (C-7) and one mono-oxygen-connected carbon at *δ*_C_ 79.4 (C-1)), with ten assigned to the aglycon part, and six to a pyranose moiety. The sugar moiety which was consistent with d-glucose underwent GC analysis of the derivatives of its hydrolysis product and was compared with a referenced d-glucose.

**Figure 2:**
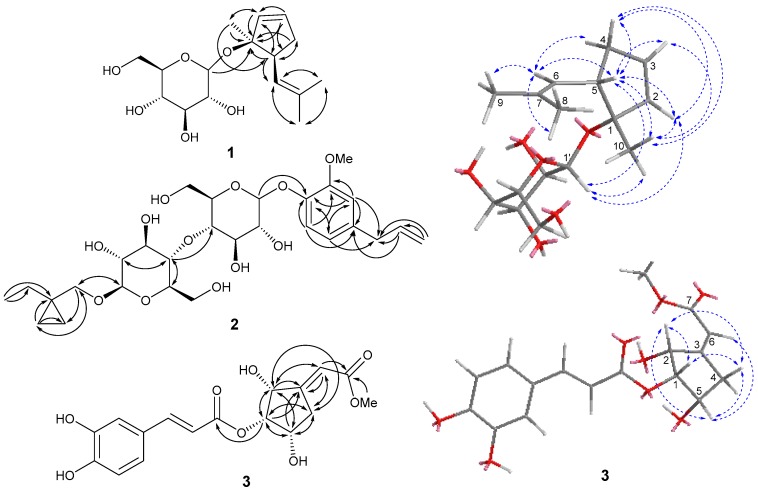
Key heteronuclear multiple-bond correlation (HMBC) (H
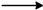
C) correlations of **1**–**3** (**left**), and key NOESY (H
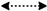
H) correlations of **1** and **3** (**right**).

The 2D NMR spectroscopic data of **1** was analyzed in detail to determine the 2D structure of the aglycone part. Starting from the typical olefinic proton of H-2, the ^1^H ^1^H-COSY cross-peaks of H-2/H-3/H-4 (*δ*_H_ 1.96)/H-5 (Figure 1), coupled with the significant HMBC correlations of H-2/C-1 and C-5; H-3/C-1, C-2, and C-5; H-4/C-1 and C-5; H-5/C-1, C-2 and C-4 suggested the presence of a cyclopentene moiety (C-1 to C-5) in **1**. The remaining signals for the aglycone part were speculated as a 2-methylprop-1-ene moiety (C-6 to C-9) on the basis of NMR data and the correlations from H-6 to C-7, Me-8 and Me-9; from Me-8 to C-6, C-7 and Me-9; from Me-9 to C-6, C-7 and Me-8 in the HMBC spectrum. Additionally, ^1^H ^1^H-COSY cross-peak of H-5/H-6 and HMBC correlations between: H-5 to C-6 and also H-6 to C-4 (Figure 1 and Figure 2), demonstrated the two moieties were fused through C-5/C-6 linkage. The position of Me-10 was assigned at C-1 and was deduced by HMBC correlations from Me-10 to C-1, C-2 and C-5 (Figure 2). Thus, the planar structure of the aglycone part for **1** was determined as in Figure 1. The location the sugar unit was thus unequivocally established to be at C-1 by the key HMBC correlation data from H-1′_glc_ to C-1 (*δ*_C_ 79.4) (Figure 2).

The relative configurations of **1** at C-1 and C-5 positions were unambiguously assigned by interpretation of the observed NOESY correlations (Figure 2). The relative 5α and Me-10α configurations were determined by the NOESY correlations from H-5 to H-4, H-6, Me-10 and H-1′; from Me-10 to H-2, H-3, H-4, H-5 and H-1′. Finally, compound **1** was identified as (*rel*)-(1*R*, 5*R*)-1-methyl-5-(2-methylprop-1-en-1-yl)cyclopent-2-en-1-*O*-*β*-d-glucopyranosideby using a molecular modeling program called Chem 3D Ultra 14.0,which used MM2 force field calculations for energy minimization to provide a detailed computer-modeled 3D structure.

Compound **2** was obtained as light-yellow amorphous powder. Its molecular formula was established as C_28_H_42_O_12_ from its ^13^C-NMR data and the protonated ion peak at *m*/*z* 571.2798 [M + H]^+^ in the HRESIMS (positive ion mode) spectrum (calcd for C_28_H_43_O_12_, 571.2749), representing eight degrees of unsaturation. The ^1^H-NMR spectrum of **2** (Table 1) exhibited signals classified as one typical ABX coupling system of a 1,2,4-trisubstituted benzene at *δ*_H_ 6.80 (1H, d, *J* = 1.8 Hz, H-3), 6.67 (1H, dd, *J* = 1.8, 8.4 Hz, H-5), and 7.00 (1H, d, *J* = 8.4 Hz, H-6), one terminal double bond at *δ*_H_ 5.94 (1H, m, H-8) and 5.03–5.08 (2H, m, H-9), one methoxy at *δ*_H_ 3.74 (3H, s, OMe-2) and one methyl at *δ*_H_ 0.87 (3H, t, *J* = 7.2 Hz, Me-6‴). In addition, two obvious anomeric protons at *δ*_H_ 4.84 (1H, d, *J* = 7.2 Hz, H-1′) and 4.09 (1H, d, *J* = 7.8 Hz, H-1″) which correlated with carbon signals at *δ*_C_ 100.2 (C-1′) and 102.8 (C-1″) observed in the HSQC spectrum, indicated two pyranose moieties. The relatively large coupling constants of anomeric protons indicated that the two pyranose moieties possessed *β*-linkages. The ^13^C-NMR and HSQC spectra of **2** showed twenty-eight carbon signals (Table 1), classified into two methyls (including one methoxy at *δ*_C_ 55.6 (OMe-2)), eight methylenes (including two oxygenated methylenes at *δ*_C_ 60.8 (C-6′) and 61.1 (C-6″), one terminal double bond at *δ*_C_ 115.49 (C-9), and six high field methylenes), fourteen methines (including one olefinic carbon at *δ*_C_ 137.9 (C-8), three phenyl carbons at *δ*_C_ 112.9 (C-3), 120.8 (C-5), 115.53 (C-6), eight oxygenated methines, and two anomeric carbons at *δ*_C_ 100.2 (C-1′) and 102.8 (C-1″)), and four quaternary carbons (including three phenyl carbons at *δ*_C_ 144.86 (C-1), 144.87 (C-2) and 133.4 (C-4)), with twelve to two pyranose units. The two pyranose units were identified as d-glucose by means of the same method described above. Analysis of the ^1^H and ^13^C-NMR data of **2** revealed a strong similarity to that of 2,6-dimethoxy-4-(prop-2-enyl) phenyl *O*-α-l-rhamnopyranosyl-(1→6)-*β*-d-glucopyranoside [14], except for the differences in the phenyl and sugar chain moieties. Preliminary inspection of the NMR data of the phenyl moiety revealed the presence of only one methyl at C-2, instead of two methyls at C-2 and C-6 in 2,6-dimethoxy-4-(prop-2-enyl) phenyl *O*-α-l-rhamnopyranosyl-(1→6)-*β*-d-glucopyranoside as established by the typical ABX system of **2** and the significant long range correlations of H-3/C-1, C-2, C-4, C-5, and C-7; H-5/C-1, C-3, and C-7; H-6/C-1, C-2, and C-4 in the HMBC spectrum (Figure 2). Detailed analysis of the sugar chain of **2** indicated that two *β*-d-glucoses in **2** linked through a different C-4′/C-4″ linking mode, which was consolidated by the HMBC correlations of H-4″/C-4′, C-2″, and C-5″, H-2″ and H-5″/C-4″. With respect to the linking mode of the sugar chain, one additional (1-ethylcyclopropyl) methanol group was assigned at C-1″ of the outer *β*-d-glucose, which were confirmed by the HMBC correlations of H-1‴a/C-1″, C-3‴, and C-4‴, H-1‴b/C-1″, C-3‴, and C-4‴, H-4‴a/C-1‴, C-2‴, and C-3‴, H-6‴/C-2‴ and C-5‴. The substitution site of the sugar chain at C-1 was thus unequivocally established by key HMBC correlation peaks from H-1′_glc_ to C-1 (*δ*_C_ 144.86) (Figure 2). Finally, compound **2** was identified as 2-methoxy-4-(prop-2-enyl)-1″-(1-ethylcyclopropyl) methanol phenyl *O*-*β*-d-glucopyranosyl-(4→4)-*β*-d-glucopyranoside.

Compound **3** was obtained as a brown gum. The molecular formula of C_17_H_18_O_8_ was established by its ^13^C-NMR and (+) HRESIMS spectrum, which provided an ion peak at *m*/*z* 373.0953 [M + Na]^+^ (calcd for C_17_H_18_NaO_8_, 373.0894), representing nine degrees of unsaturation. With the aid of ^13^C-NMR and HSQC spectra, the seventeen carbons (Table 1) were classified into one methoxy at *δ*_C_ 52.3 (OMe-7), one methylene, nine methines (including three aromatic carbons at *δ*_C_ 115.3 (C-2′), 116.2 (C-5′) and 121.9 (C-6′), three olefinic methines at *δ*_C_ 140.1 (C-6), 145.9 (C-7′), and 114.4 (C-8′), and three oxygenated methines at *δ*_C_ 70.2 (C-1), 68.0 (C-2), and 65.8 (C-5)), six quaternary carbons (including two ester carbonyls at *δ*_C_ 166.7 (C-7) and 166.5 (C-9′), three aromatic carbons at *δ*_C_ 125.9 (C-1′), 146.0 (C-3′), and 148.9 (C-4′), and one olefinic carbon at *δ*_C_ 140.1 (C-6)). The proton and carbon NMR data of **3** were very similar to those of 1-(3′,4′-dihydroxylcinnamoyl)-cyclopenta-2,5-diol [15], with the exception of one additional methyl acetate group connected with C-3 via C_3_=C_6_ double bond, which was further proved by a combined interpretation of HMBC correlations of H-1 (*δ*_H_ 5.10)/C-2, C-3, C-4, C-5, C-9′, H-2 (*δ*_H_ 3.78)/C-1, C-2, C-3, C-6, H_2_-4 (*δ*_H_ 2.65-2.69, 2.21–2.25)/C-1, C-2, C-4, C-5, C-6, H-5 (*δ*_H_ 4.28)/C-1, C-3, C-4, C-5, H-6 (*δ*_H_ 6.72) with C-2, C-3, C-4, C-7, OMe (*δ*_H_ 3.69)/C-7 (Figure 2).

The relative configuration of **3** was determined as shown in Figure 2 by comprehensive analysis of the NOESY correlations and coupling constants. The NOE correlations of H-1/H-2, H-4a, H-4b; H-2/H-1, H-4b, H-5; H-5/H-4a, H-6 (Figure 2b), banded with the small coupling constants of H-1 with H-2 (*J* = 6.6 Hz) and H-1 with H-5 (*J* = 4.8 Hz), demonstrated the *β*-orientations for H-1, H-2 and H-5, and *α*-orientations for OH-2 and OH-5. In addition, the Z form of the olefinic geometries of C-3/C-6 was deduced by the NOE correlation of H-6/H-5. The absolute configuration of **3** was determined by comparing its experimental ECD spectrum with that of (3*S*,5*R*,6*S*,7*E*)3,5,6-trihydroxy-7-megastigmen-9-one [16,17], where the curve trend of **3** displayed a negative cotton effect at 222 nm consistent with the 2*S* enantiomer. Consequently, compound **3** was assigned 1*S*, 2*S*, and 5*S* configurations based on the above mentioned NOESY correlations, and the structure was elucidated as (*Z*)-(1*S*,2*S*,5*S*)-2,5-dihydroxy-3-(2-methoxy-2-oxoethylidene)cyclopentyl 3-(3,4-dihydroxyphenyl) acrylate.

**Figure 3:**
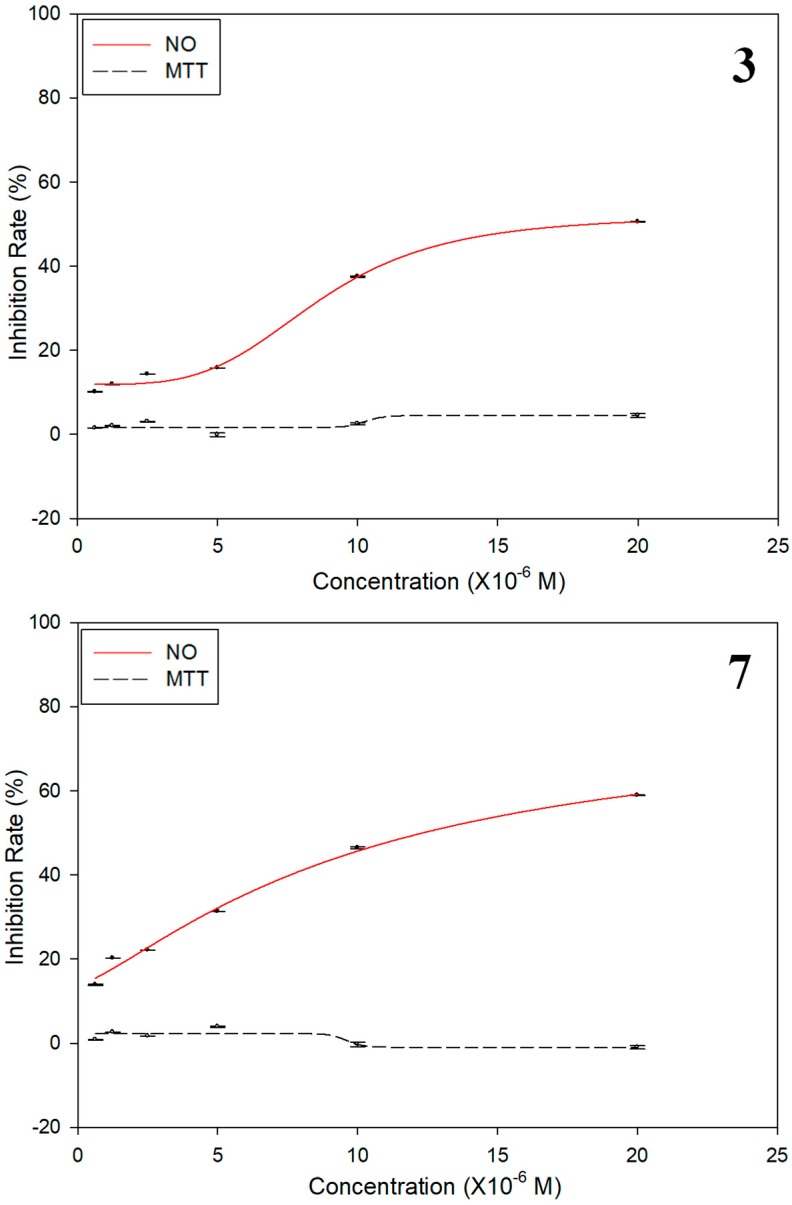
IC_50_ values of compounds **3** and **7** on NO production in LPS-induced Raw 264.7 cells. (Raw 264.7 cells were subjected to compounds **3** and **7** with six different concentrations and incubated for 2 h firstly, and then stimulated by adding LPS for 24 h. Then, Griess reagents I and II were added to cell supernatants to detect the NO production levels. The cytotoxicity against macrophage Raw 264.7 cells of compounds **3** and **7** were investigated using the MTT method; NO: Inhibition rates of NO release (%); MTT: Inhibition rates of cell viability (%)).

The anti-inflammatory activities of compounds **1**–**7** were evaluated against nitric oxide (NO) production in an in vitro inflammatory model of macrophage Raw 264.7 cells because the application of *S*. *officinalis* as a folk medicinal plant concerns the treatment of inflammation. The results of the bioassay were shown in Table 2 and Figure 3, which showed that compounds **3** and **7** exhibited moderate inhibitory effects on NO production with IC_50_ values of 12.2 ± 0.8 and 18.9 ± 0.3 μM, respectively. However, the anti-inflammatory activities of compounds **1**, **2**, **4**, **5**, and **6** against nitric oxide production were weaker compared with the positive control dexamethasone (2.2 ± 0.02 μM). Other compounds were inactive (IC_50_ > 100 μM). In addition, the cytotoxicity against macrophage Raw 264.7 cells of compounds **1**–**7** were also investigated using the MTT method, and none showed any cell toxicity at a concentration of 20 μM (Table 2). In conclusion, we can state that *S*. *officinalis* is an interesting source of active compounds. The hydroxyl substituted benzene ring and conjugated ester moiety might take significant effect for anti-inflammatory activity.

## 3. Materials and Methods

### 3.1. General

Optical rotations were determined using a SEEWE SGW-2 digital polarimeter. ECD data were recorded on an Applied Photophysics Chirascan ECD spectrometer. An HRESI-MS analysis was obtained using a Bruker Impact II mass spectrometer. The 1D and 2D NMR spectra were taken on either a BURKER 400 NMR or a Varian 600 NMR spectrometer. The preparative HPLC experiments were conducted on a Shimadzu LC-6AD packed with an SPD-10A detector and a reversed-phase C_18_ column (Shim-pack PREP-ODS (H). Kit, 20 × 250 mm, 5 μm). Silica gel (200−300 mesh, Qingdao Haiyang Chemical Co. Ltd., Qingdao, China), and ODS (45 μm, YMC Co. Ltd., Kyoto, Japan) were used for column chromatography (CC). HPLC grade Acetonitrile (MeCN) was used for preparative HPLC experiments, and other solvents were of analytical grade.

### 3.2. Plant Material

The origin of *S*. *officinalis* was identical to that mentioned in our previous studies [8,9].

### 3.3. Extraction and Isolation

Air-dried and pulverized stems of *S*. *officinalis* (10 Kg) were extracted with 95% EtOH three times (2 h, 1 h, 1 h) under reflux conditions, and the condensed extracts were successively subjected to multi-solvent partition with petroleum ether, EtOAc, and *n*-BuOH sequentially as previously described [6]. The EtOAc fraction was subjected to CC over silica gel fractionation to furnish eleven fractions (designated as fractions 1 to 11). Fraction 4 (3.0 g, eluted with CH_2_Cl_2_/MeOH, 15:1) was fractioned by a Flash C_18_ column eluted with gradient elution of MeOH/H_2_O (5%→100%) to yield five subfractions (F4-1-F4-5). Fraction 4-3 (2.5 g, eluted with 12% MeOH/H_2_O) was further chromatographed on a Flash C_18_ column by gradient elution of MeOH/H_2_O (5%→100%) to give compound **4** (22 mg, tR = 5.689 min, purity of 97% by HPLC analysis) and compound **5** (17 mg, tR = 10.000 min, purity of 96% by HPLC analysis). Fraction 5 (10.6 g, eluted with CH_2_Cl_2_/MeOH, 15:1) was separated on a Flash C_18_ column with gradient elution of MeOH/H_2_O (12%→100%) to give four subfractions (F5-1-F5-4). Fraction 5-2 (2.1 g, eluted with 45% MeOH/H_2_O) was further chromatographed to give ten subfractions (F5-2-1-F5-2-10), using a Flash C_18_ column by gradient elution of MeOH/H_2_O (5%→100%). Fraction 5-2-5 (60 mg, eluted with 45% MeOH/H_2_O) was further purified using preparative HPLC (mobile phase: MeCN/H_2_O (20:80, *v/v*); flow rate: 3 mL min^−1^; UV detection at 210 nm) to obtain compound **2** (4 mg, tR = 20.600 min, purity of 91% by HPLC analysis) and compound **6** (10 mg, tR = 14.248 min, purity of 92% by HPLC analysis), respectively. Fraction 5-3 (1.2 g, eluted with 60% MeOH/H_2_O) was further fractioned to give ten subfractions (F5-3-1-F5-3-10) by a Flash C_18_ column eluted with MeOH/H_2_O (5%→100%). Compound **1** (12 mg, tR = 12.970 min, purity of 93% by HPLC analysis) was purified using preparative HPLC system [mobile phase: MeCN/H_2_O (30:70, *v/v*); flow rate: 3 mL min^−1^; UV detection at 210 nm] from Fraction 5-3-9 (60 mg, eluted with 60% MeOH/H_2_O). Fraction 6 (5.0 g, eluted with CH_2_Cl_2_/MeOH, 15:1) was chromatographed on a Flash C_18_ column with a MeOH/H_2_O gradient (5%→100%) to give nine subfractions (F6-1-F6-9). Compound **7** (26 mg, tR = 10.189 min, purity of 95% by HPLC analysis) was purified using preparative HPLC system [mobile phase: MeCN/H_2_O (12:88, *v/v*); flow rate: 3 mL min^−1^; UV detection at 210 nm] from fraction 6-1 (100 mg, eluted with 5% MeOH/H_2_O). Fraction 7 (5.0 g, eluted with CH_2_Cl_2_/MeOH, 10:1) was separated using Flash C_18_ CC with a MeOH/H_2_O gradient (5%→100%) to give six subfractions (F7-1-F7-6). Fraction 7-1 (0.8 g, eluted with 12% MeOH/H_2_O) was further chromatographed by CC over Sephadex LH-20 with MeOH as mobile phase to give five subfractions (F7-1-1-F7-1-5). Compound **3** (12 mg, tR = 25.476 min, purity of 91% by HPLC analysis) was purified using preparative HPLC system (mobile phase: MeCN/H_2_O (18:82, *v/v*); flow rate: 3 mL min^−1^; UV detection at 285 nm) from fraction 7-1-5 (80 mg).

*(rel)-(1R, 5R)-1-Methyl-5-(2-methylprop-1-en-1-yl)cyclopent-2-en-1-O-β-d-glucopyranoside* (**1**): brown, gum; ${\left[\alpha \right]}_{D}^{20}$ − 25.0 (*c* 0.067, MeOH). ^1^H (DMSO-*d*_6_, 400 MHz) and ^13^C-NMR (DMSO-*d*_6_, 100 MHz), see Table 1; HRESIMS (positive-ion mode): *m*/*z* 337.1802 [M + Na]^+^ (calcd for C_16_H_26_NaO_6_, 337.1622).

*2-Methoxy-4-(prop-2-enyl)-1″-(1-ethylcyclopropyl) methanol phenyl O-β-d-glucopyranosyl-(4→4)-β-d-glucopyranoside* (**2**): light yellow, amorphous powder; ${\left[\alpha \right]}_{D}^{20}$ − 23.7 (*c* 0.09, MeOH). ^1^H (DMSO-*d*_6_, 600 MHz) and ^13^C-NMR (DMSO-*d*_6_, 150 MHz), see Table 1; HRESIMS (positive-ion mode): *m*/*z* 571.2798 [M + H]^+^ (calcd for C_28_H_43_O_12_, 571.2749).

*(Z)-(1S,2S,5S)-2,5-Dihydroxy-3-(2-methoxy-2-oxoethylidene)cyclopentyl 3-(3,4-dihydroxyphenyl) acrylate* (**3**): brown, gum; ${\left[\alpha \right]}_{D}^{20}$ − 34.1 (*c* 0.27, MeOH). ^1^H (DMSO-*d*_6_, 600 MHz) and ^13^C-NMR (DMSO-*d*_6_, 150 MHz), see Table 1; HRESIMS (positive–ion mode): *m*/*z* 373.1013 [M + Na]^+^ (calcd for C_17_H_18_NaO_8_, 373.0894).

### 3.4. Acid Hydrolysis of Compounds ***1*** and ***2*** to Determine the Absolute Configuration of the Monosaccharides

Compounds **1** and **2** were subjected to acid hydrolysis the same as described in our previous study [8], as well as the determination of absolute sugar moiety configurations. The hydrolyzed aqueous fraction was further detected and identified as glucose comparing with the authentic glucose sample by thin layer plate analysis, using CHCl_3_-CH_3_OH-H_2_O (15:9:2) as a developing solvent and 10% H_2_SO_4_ in 95% EtOH as a detection reagent. Then, the methylated thiazolidine derivatives of the hydrolyzed sugar moieties and the corresponding authentic glucose were prepared, and their absolute configurations were further identified by comparing the retention times of the hydrolyzed sugar derivatives with that of the authentic glucose derivative in the GC analysis (conditions in the experiment were as follows: DB-5 column, 30 m × 250 μm × 0.25 μm; FID detection; carrier gas of N_2_; injection temperature of 250 °C, detection temperature of 260 °C, column temperature of 60 °C (0 min) raised up to 300 °C in 24 min by 10 °C/min, and 300 °C kept for 10 min. tR d-glucose 21.10 min).

### 3.5. Anti-Inflammatory Activity Assay Against NO Production

The anti-inflammatory activity assay against NO production in RAW 264.7 mouse peritoneal macrophages was conducted mainly using the procedures described previously [18]. The macrophage Raw 264.7 cells were subjected to dexamethasone (Sigma, St. Louis, MO, USA, >98%) and isolated compounds (**1**–**7**) with six concentrations of 0.63, 1.25, 2.5, 5.0, 10 and 20 μM and incubated for 2 h firstly, and then stimulated by adding lipoplysaccharide (LPS, 1.0 μg/mL). After being stimulated for 24 h, 50 μL of cell supernatants were taken out from each well and mixed with an equal volume of Griess reagents I and II to detect the NO production levels by measuring absorbance at 570 nm. All experiments were performed in triplicate. The NO inhibition is calculated as the percentage reduction of LPS stimulation.

The cytotoxicity against macrophage Raw 264.7 cells was investigated using the MTT assay [9]. Briefly, after the Raw 264.7 cells were treated with the test compounds for 24 h, 50 μL of MTT (5 mg/mL) was added to each well, and the cells were incubated for an additional 4 h. After the formazan crystals formed, cell supernatants were removed and 100 μL of DMSO was added to dissolve the crystals. Absorbance for each well was analyzed in the same way as that for NO inhibition assay.

## 4. Conclusions

In the present study, one new monoterpene glycoside (**1**), one new phenyl glycoside (**2**), one new caffeoyl derivative (**3**), and four known compounds (**4**–**7**) were isolated from *S. officinalis*. This is the first time that these kinds of compounds are reported in the title plant. Compounds **3** and **7** exhibited moderate inhibitory effects on NO production, and neither showed any cell toxicity against macrophage Raw 264.7 cells. Meanwhile, none of the other five compounds showed any anti-inflammatory activities. These definite pharmacological findings indicated limited structure–activity relationships as hydroxyl substituted benzene rings and conjugated ester moieties might take significant effect for anti-inflammatory activity, supported the application of the *S. officinalis* in Miao ethnomedicine system to treat inflammation.

## Figures and Tables

**Table 1:** ^1^H and ^13^C-NMR spectroscopic data of **1**–**3** (in DMSO-*d*_6_, TMS) (*δ* in ppm, *J* in Hz).

Positions	1 *^a^*		2 *^b^*		3 *^b^*	
1		79.4		144.86	5.10 dd (4.8,6.6)	70.2
2	5.98 dd (11.2, 17.6)	144.1		144.87	3.78 dd (4.2, 6.6)	68.0
3	5.09 overlapped	114.1	6.80 d (1.8)	112.9		127.4
4a	1.96 m	22.6		133.4	2.65–2.69 m	27.9
4b					2.21–2.25 m	
5	1.50 m	40.2	6.67 dd (1.8, 8.4)	120.8	4.28 m	65.8
6	5.09 overlapped	125.3	7.00 d (8.4)	115.53	6.72 brs	140.1
7		130.8	3.30 d (6.6)	39.1		166.7
8	1.61 s	25.9	5.94 m	137.9		
9	1.54 s	18.0	5.03–5.08 m	115.49		
10	1.22 s	24.0				
1′	4.13 d (8.0)	98.2	4.84 d (7.2)	100.2		125.9
2′	2.90 m	74.0	3.23–3.28 m	73.1	7.05 d (2.0)	115.3
3′	3.09 t (8.4)	77.5	3.23–3.28 m	76.98		146.0
4′	3.02 t (9.2)	70.7	3.23–3.28 m	76.78		148.9
5′	2.95 m	77.1	3.13–3.17 m	69.7	6.76 d (8.4)	116.2
6′a	3.58 d (11.2)	61.7	3.65–3.67 m	60.8	7.01 dd (2.0, 8.4)	121.9
6′b	3.38 m		3.42–3.45 m			
7′					7.48 d (15.6)	145.9
8′					6.25 d (15.6)	114.4
9′						166.5
1″			4.09 d (7.8)	102.8		
2″			2.93 td (8.4, 4.2)	73.4		
3″			3.05–3.08 m	76.85		
4″			3.10–3.13 m	76.81		
5″			3.01–3.05 m	70.1		
6″a			3.65–3.67 m	61.1		
6″b			3.42–3.45 m			
1‴a			3.74 d (13.2)	68.5		
1‴b			3.40 d (13.2)			
2‴				31.2		
3‴			1.23–1.32 ov	25.2		
4‴a			1.49–1.53 m	29.2		
4‴b			1.23–1.32 ov			
5‴			1.23–1.32 ov	21.9		
6‴			0.87 t (7.2)	13.9		
OMe			3.74 s (OMe-2)	55.6	3.69 s	52.3

*^a^* The NMR data were recorded in DMSO-*d*_6_ at 400 MHz for *δ*_H_ and at 100 MHz for *δ*_C_. *^b^* The NMR data were recorded in DMSO-*d*_6_ at 600 MHz for *δ*_H_ and at 150 MHz for *δ*_C_.

**Table 2:** Inhibitory effects on NO production against Raw 264.7 cells of compounds **1**–**7** and their cytotoxicities ^a^.

Compounds	IC_50_ (μM)	Inhibition Rate (%) ^b^
	NO	Cytotoxicity
**1**	>100	1.8
**2**	>100	−0.5
**3**	12.2 ± 0.8	4.7
**4**	>100	1.1
**5**	>100	4.5
**6**	>100	1.2
**7**	18.9 ± 0.3	2.4
Dexamethasone	2.2 ± 0.02	NT ^c^

^a^ All results are expressed as mean ± SD; *n* = 3 for all groups. ^b^ Test at a concentration of 20 μM. ^c^ Not tested.

## Supplementary Materials

Supplementary data (NMR and MS spectroscopic data for compounds **1**–**3**) associated with this article are available online.
