# Supplementary material for: Chemical Constituents from Scindapsus officinalis (Roxb.) Schott. and Their Anti–Inflammatory Activities
Source: Molecules. 2018 Oct 9;23(10):2577. doi: 10.3390/molecules23102577 (PMC6222759; doi:10.3390/molecules23102577)
Supplement: Supplementary file 1 [file molecules-23-02577-s001.pdf]

Chemical Constituents from *Scindapsus officinalis* (Roxb.) Schott. and Their Anti–Inflammatory Activities

Hongjing Dong [1],Yanling Geng [1], Xueyong Wang[2], Xiangyun Song [1], Xiao Wang [1] and Jinqian Yu [1,*]

[1] Shandong Key Laboratory of TCM Quality Control Technology, Shandong Analysis and Test Center, Qilu University of Technology (Shandong Academy of Sciences), Jinan, 250014, P. R. China
[2] College of Chinese Mareria Medica, Beijing University of Chinese Medicine, Beijing, 100102, P. R. China
* Correspondence: yujinqian87528@126.com; Tel.: +86-0531-8260-5319

Supplementary material

Table of Contents.

no.	Content	Page
**1**	**Figure S1.** The HREIMS Spectroscopic Data of Compound **1**	S6
**2**	**Figure S2.** The ^{1}H NMR Spectrum of Compound **1** in DMSO-d_6 (400 MHz)	S7
**3**	**Figure S3.** The ^{13}C NMR Spectrum of Compound **1** in DMSO-d_6 (100 MHz)	S8
**4**	**Figure S4.** The ^{1}H–^{1}H gCOSY Spectrum of Compound **1** in DMSO-d_6 (400 MHz)	S9
**5**	**Figure S5.** The HSQC Spectrum of Compound **1** in DMSO-d_6 (400 MHz for ^{1}H)	S10
**6**	**Figure S6.** The HMBC Spectrum of Compound **1** in DMSO-d_6 (400 MHz for ^{1}H)	S11
**7**	**Figure S7.** The NOESY Spectrum of Compound **1** in DMSO-d_6 (400 MHz for ^{1}H)	S12
**8**	**Figure S8.** The HREIMS Spectroscopic Data of Compound **2**	S13
**9**	**Figure S9.** The ^{1}H NMR Spectrum of Compound **2** in DMSO-d_6 (600 MHz)	S14
**10**	**Figure S10.** The ^{13}C NMR Spectrum of Compound **2** in DMSO-d_6 (150 MHz)	S15
**11**	**Figure S11.** The HSQC Spectrum of Compound **2** in DMSO-d_6 (600 MHz for ^{1}H)	S16
**12**	**Figure S12.** The HMBC Spectrum of Compound **2** in DMSO-d_6 (600 MHz for ^{1}H)	S17
**13**	**Figure S13.** The HREIMS Spectroscopic Data of Compound **3**	S18
**14**	**Figure S14.** The ^{1}H NMR Spectrum of Compound **3** in DMSO-d_6 (600 MHz)	S19
**15**	**Figure S15.** The ^{13}C NMR Spectrum of Compound **3** in DMSO-d_6 (150 MHz)	S20
**16**	**Figure S16.** The HSQC Spectrum of Compound **3** in DMSO-d_6 (600 MHz for ^{1}H)	S21
**17**	**Figure S17.** The HMBC Spectrum of Compound **3** in DMSO-d_6 (600 MHz for ^{1}H)	S22
**18**	**Figure S18.** The NOESY Spectrum of Compound **3** in DMSO-d_6 (600 MHz)	S23
**19**	**Figure S19.** The ECD Spectrum of Compound **3** in MeOH	S24

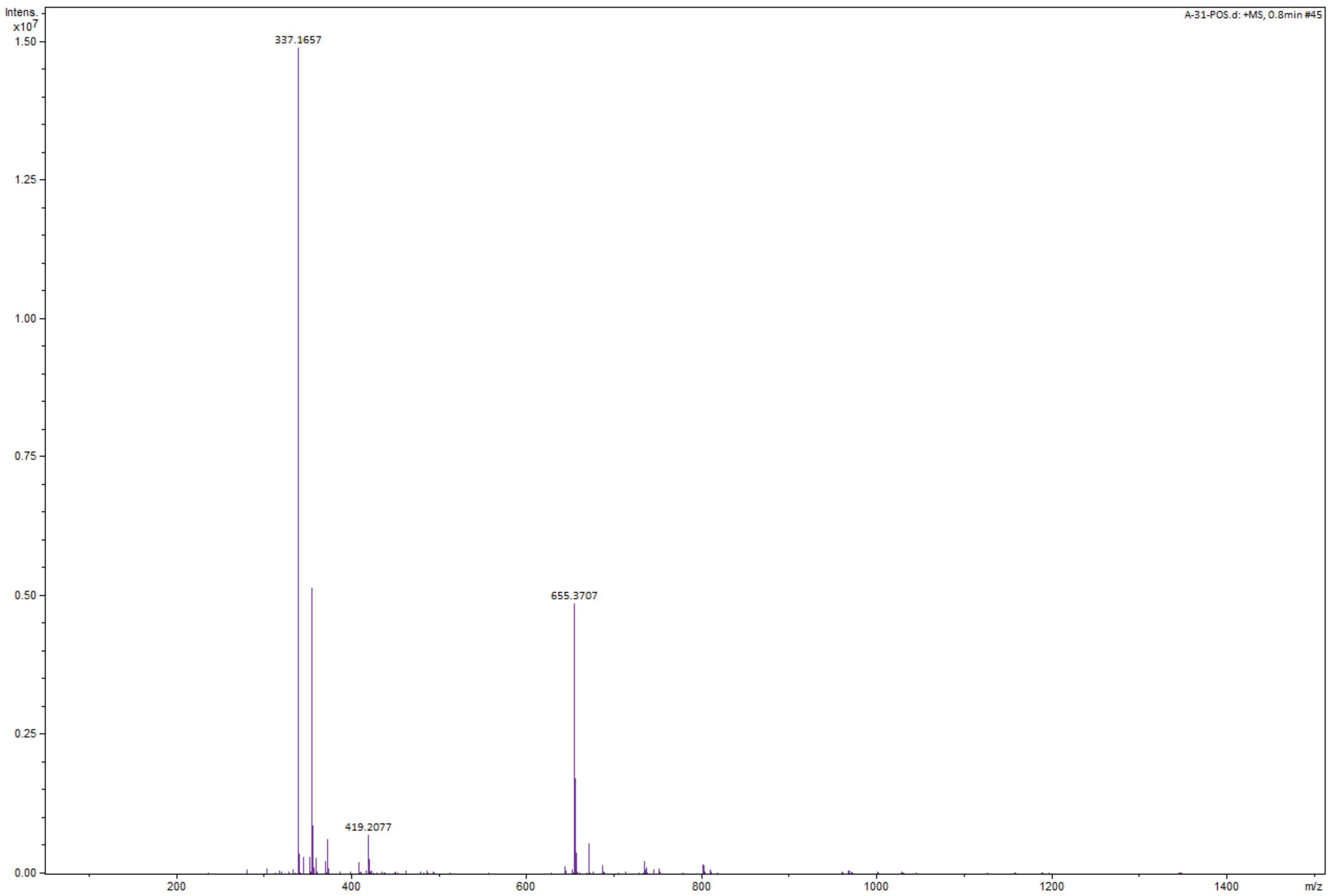

Figure S1. The HREIMS Spectroscopic Data of Compound **1.**

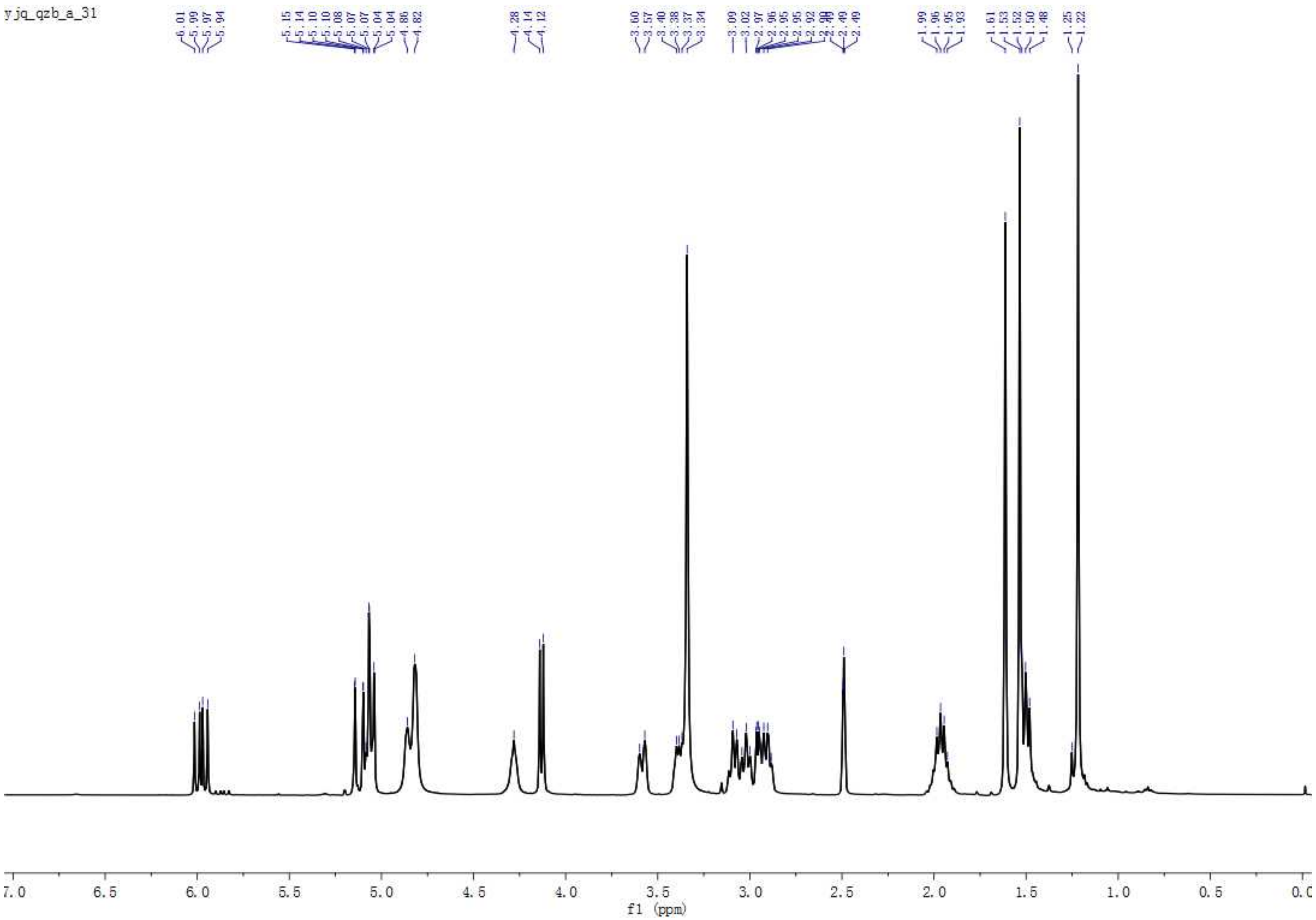

Figure S2. The ^{1}H NMR Spectrum of Compound **1** in DMSO-d_6 (400 MHz).

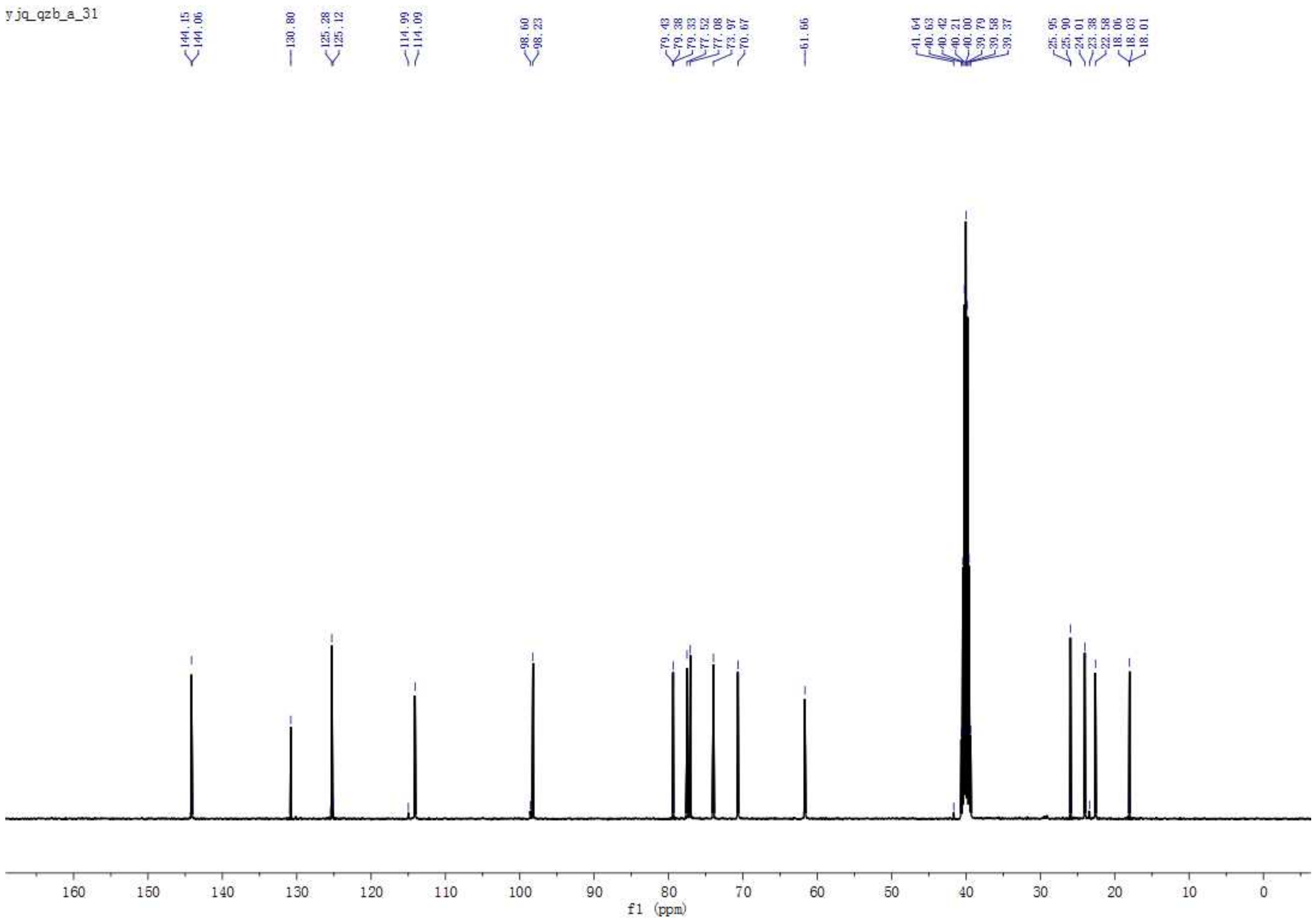

Figure S3. The ^{13}C NMR Spectrum of Compound **1** in DMSO-d_6 (100 MHz).

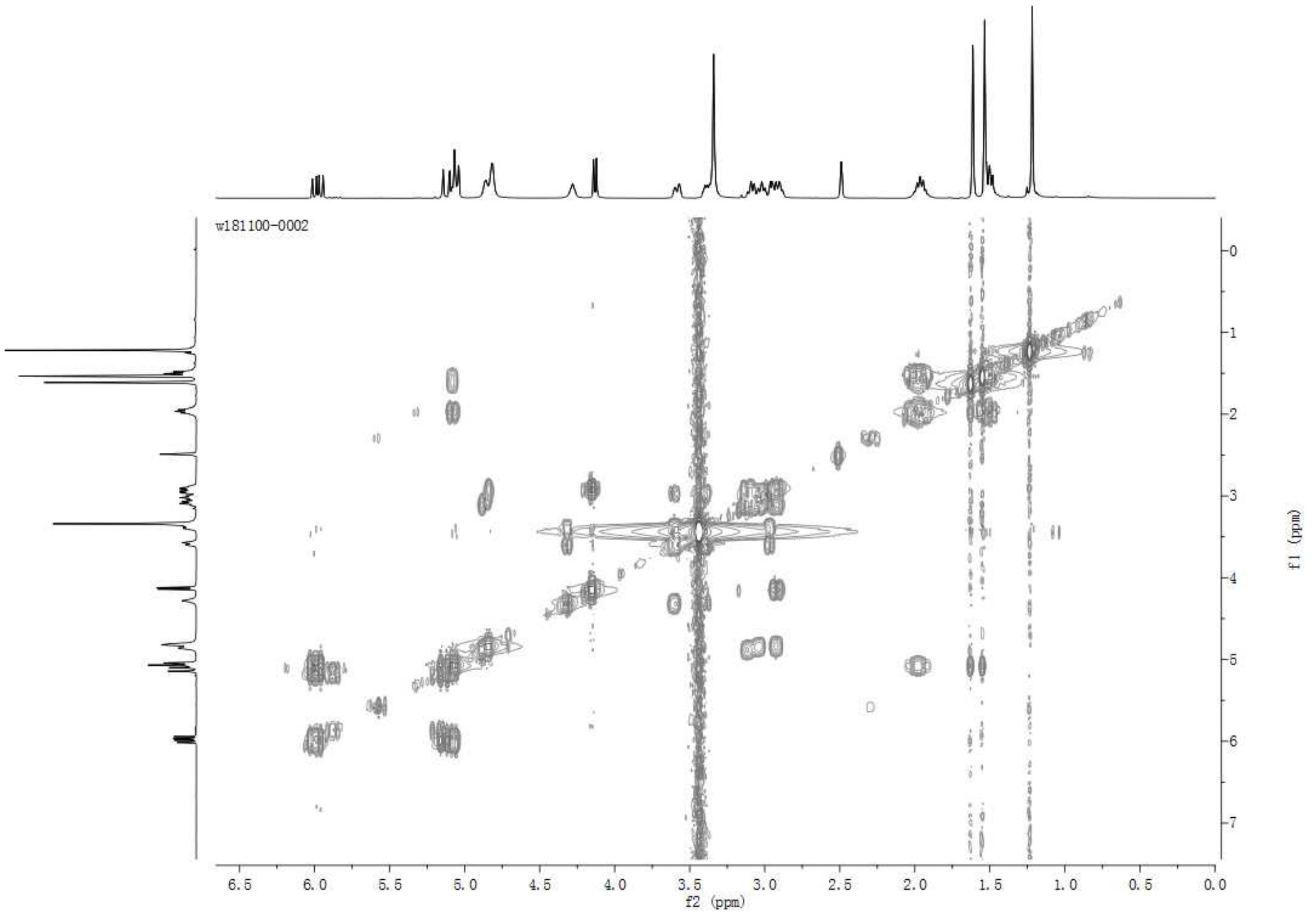

Figure S4. The ^{1}H–^{1}H gCOSY Spectrum of Compound **1** in DMSO-d_6 (400 MHz).

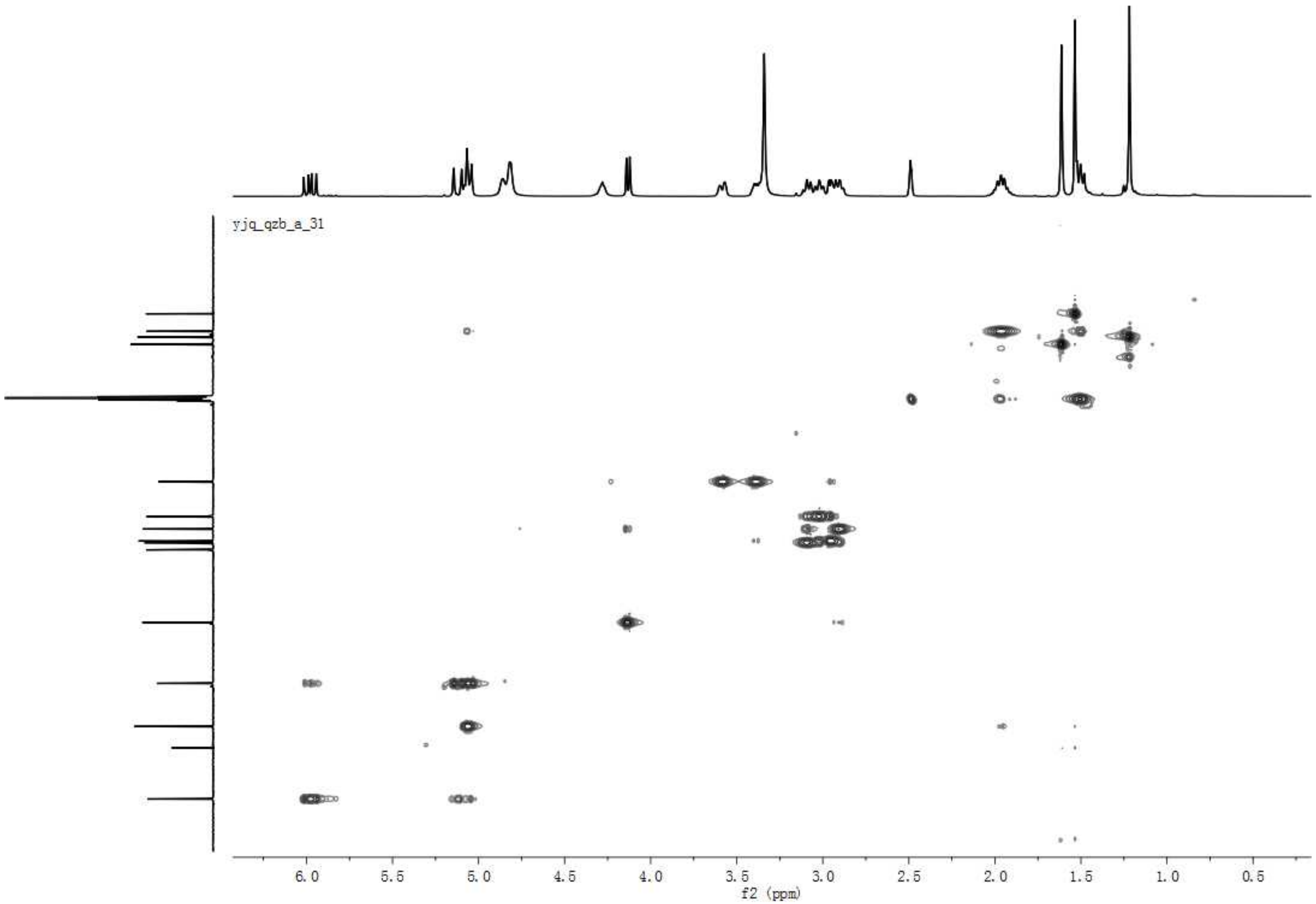

Figure S5. The HSQC Spectrum of Compound **1** in DMSO-d_6 (400 MHz for ^{1}H).

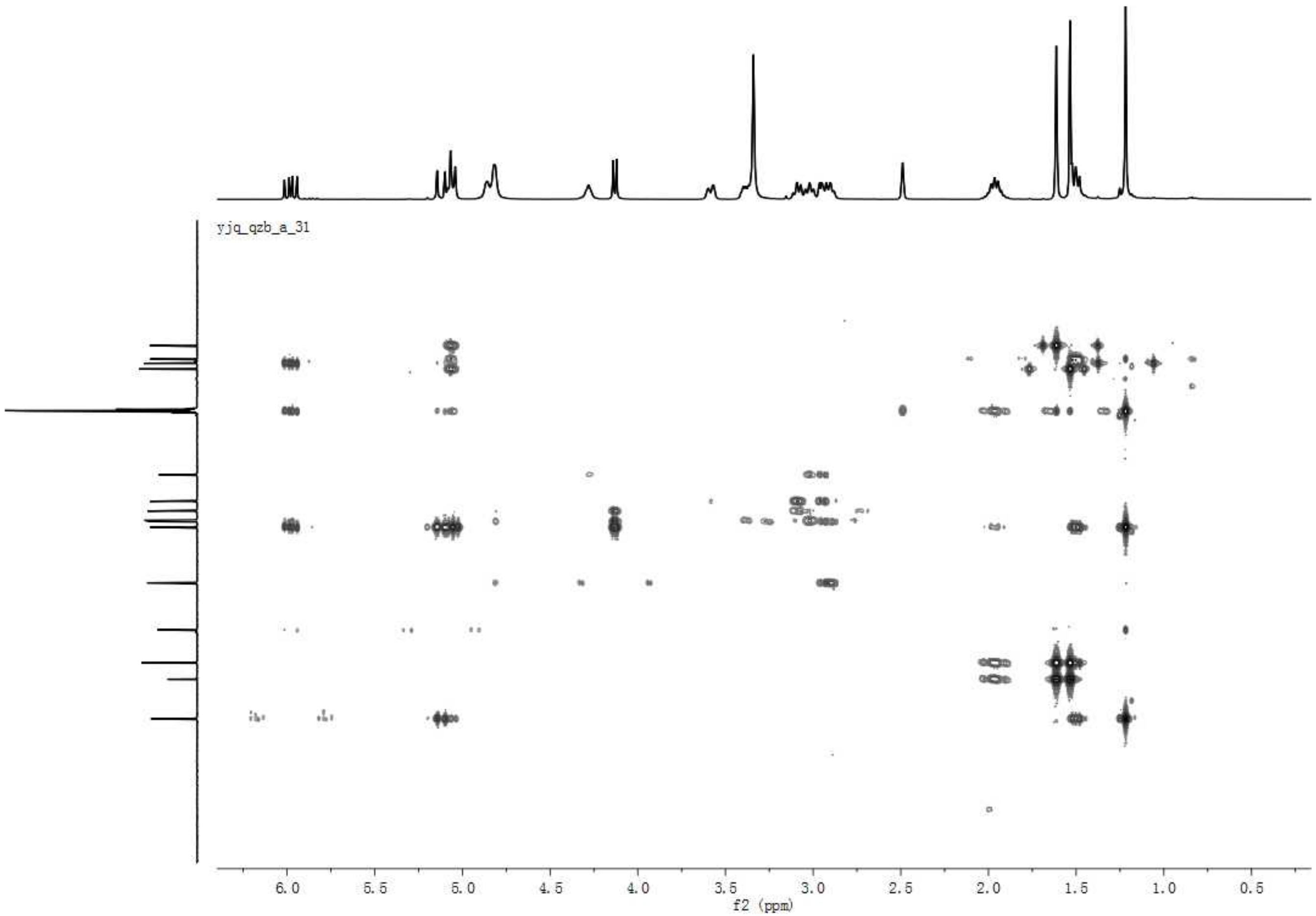

Figure S6. The HMBC Spectrum of Compound **1** in DMSO-d_6 (400 MHz for ^{1}H).

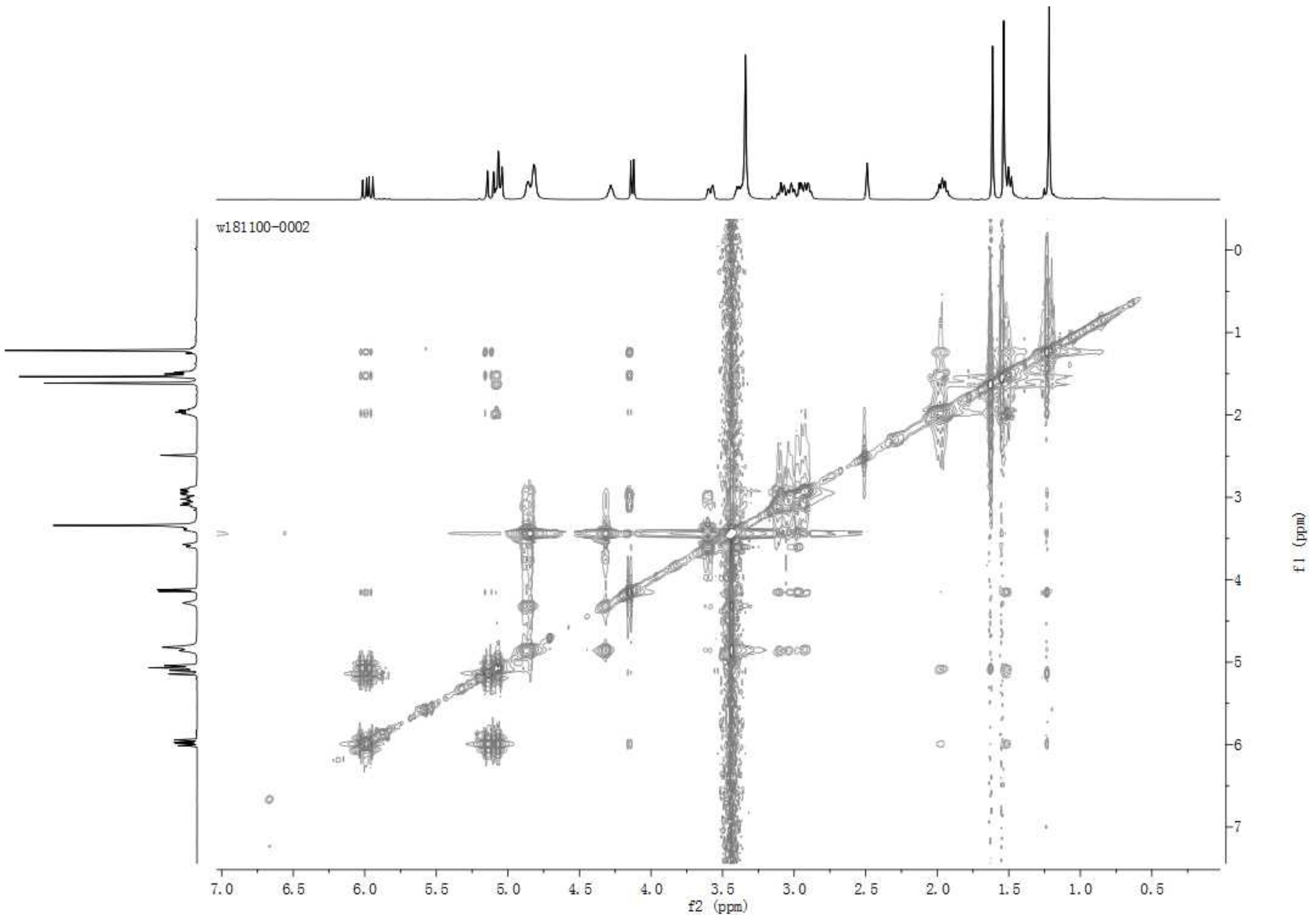

Figure S7. The NOESY Spectrum of Compound **1** in DMSO-d_6 (400 MHz for ^{1}H).

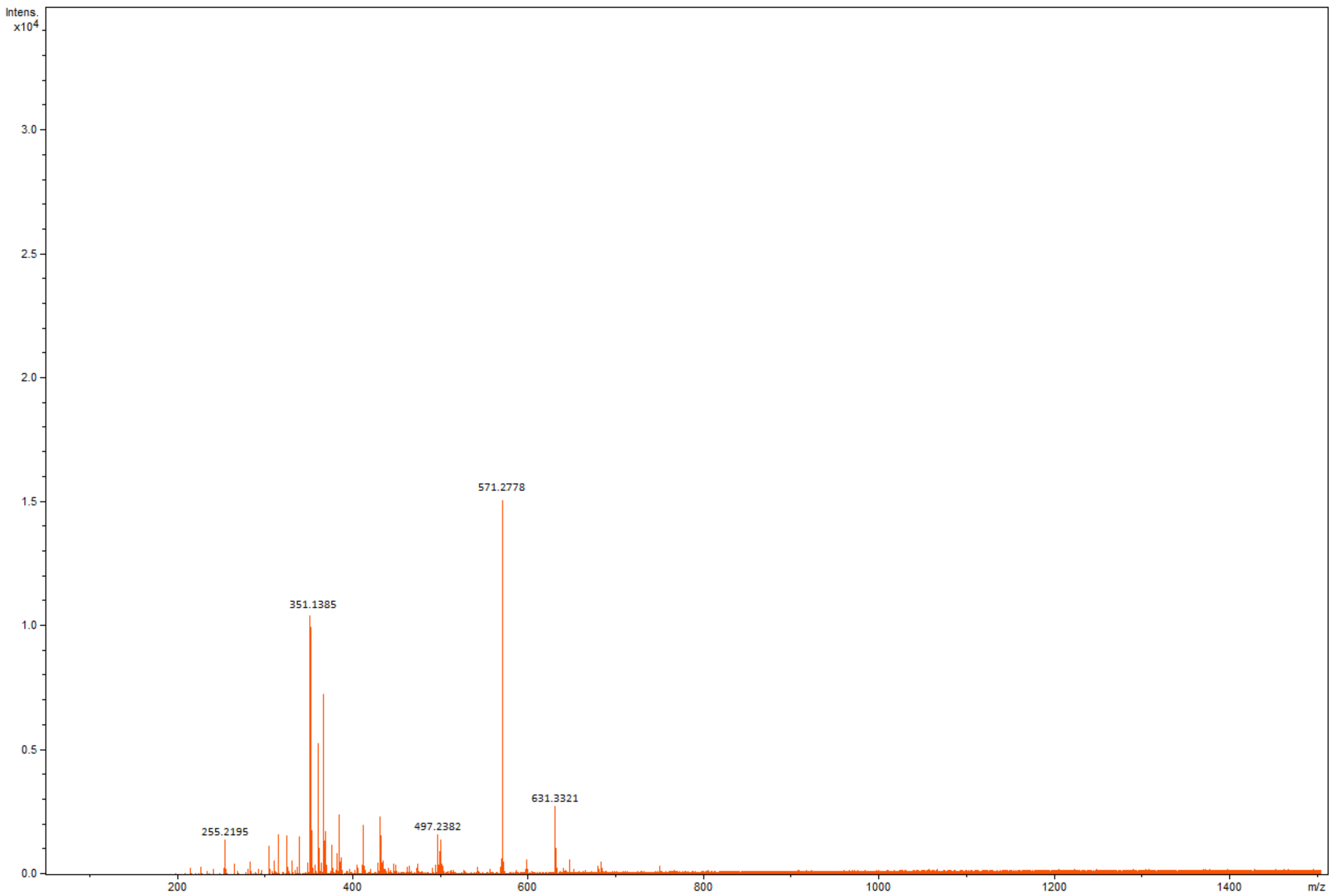

Figure S8. The HREIMS Spectroscopic Data of Compound **2.**

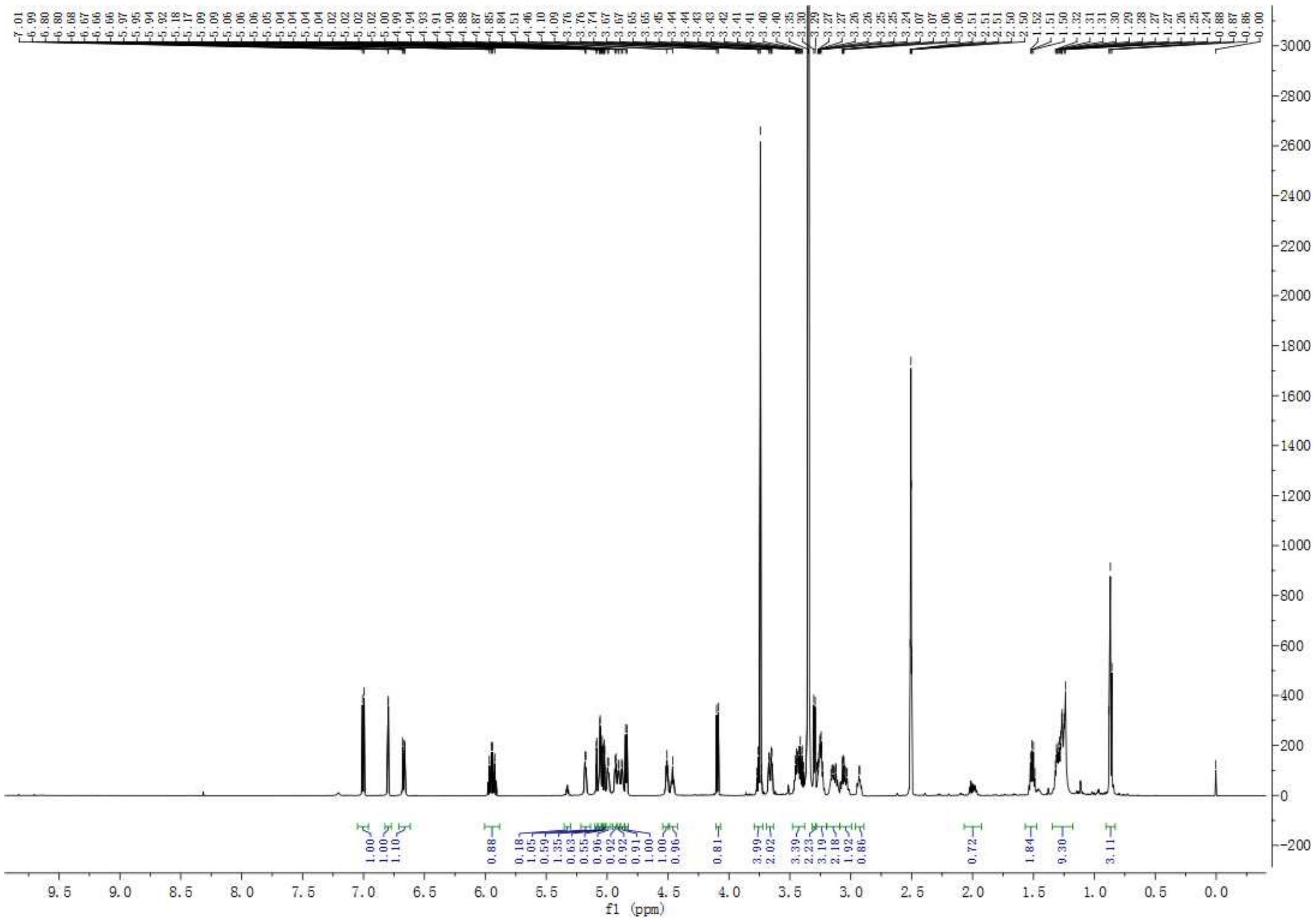

Figure S9. The ^{1}H NMR Spectrum of Compound **2** in DMSO-d_6 (600 MHz).

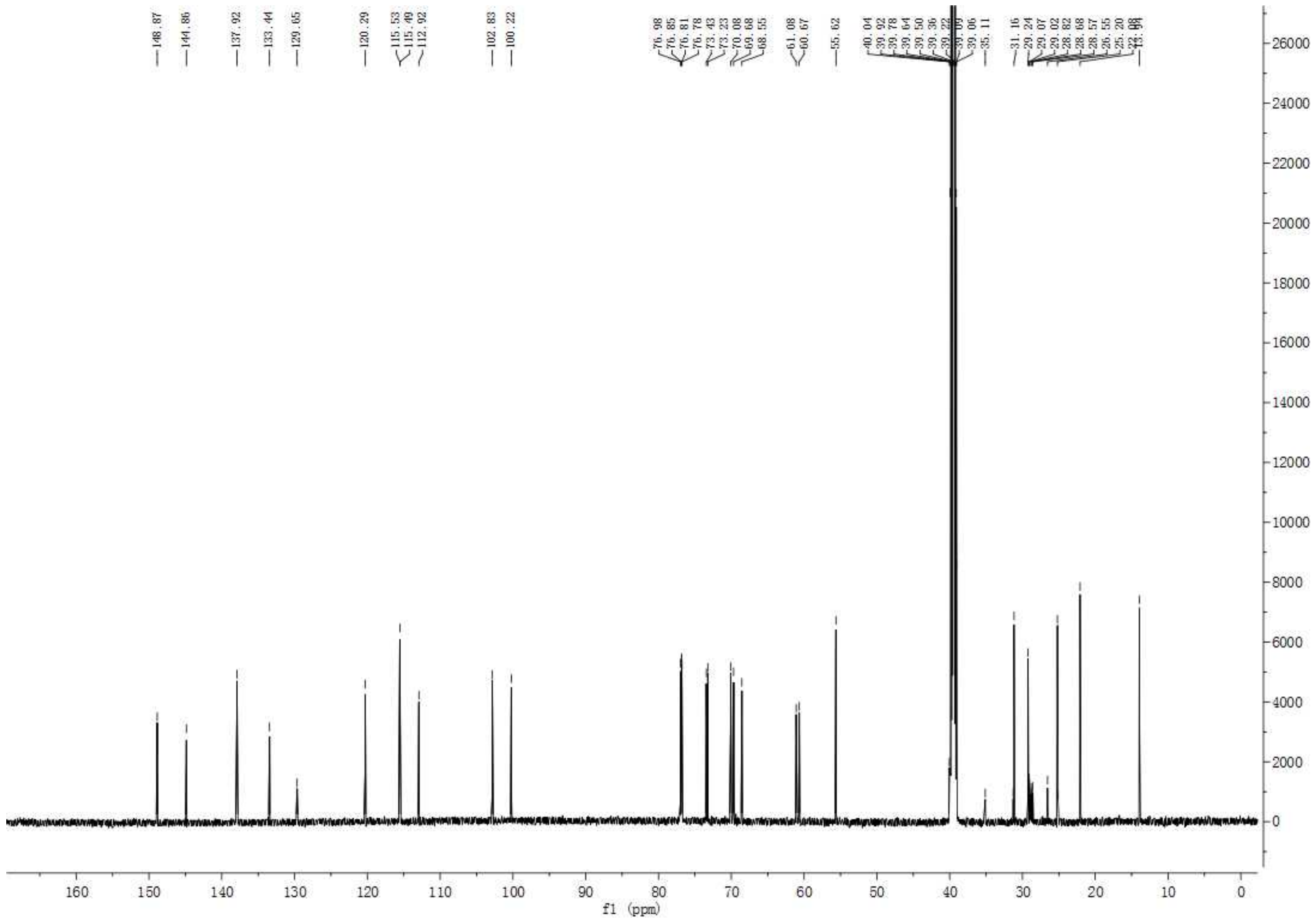

Figure S10. The ^{13}C NMR Spectrum of Compound **2** in DMSO-d_6 (150 MHz).

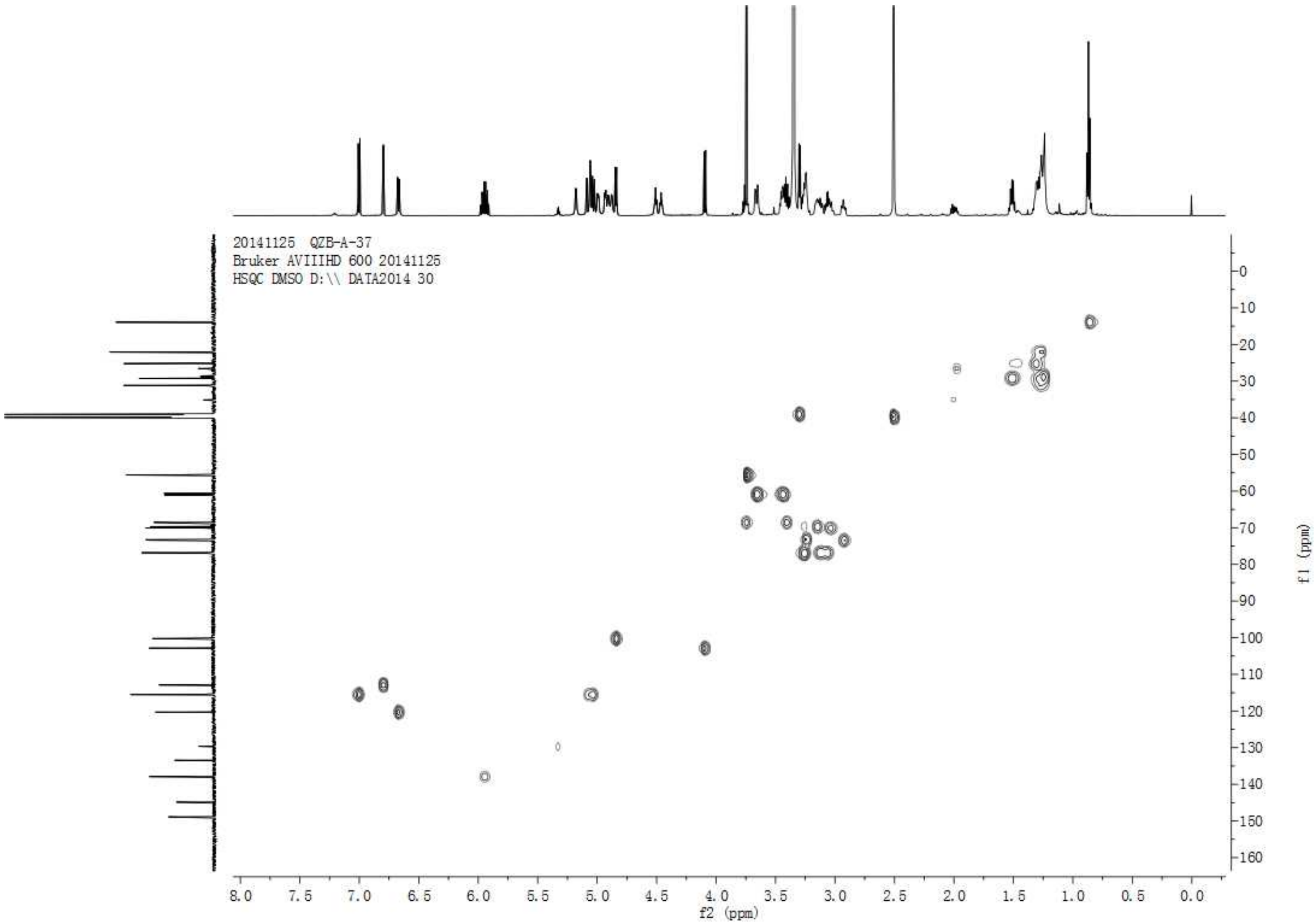

Figure S11. The HSQC Spectrum of Compound **2** in DMSO-d_6 (600 MHz for ^{1}H).

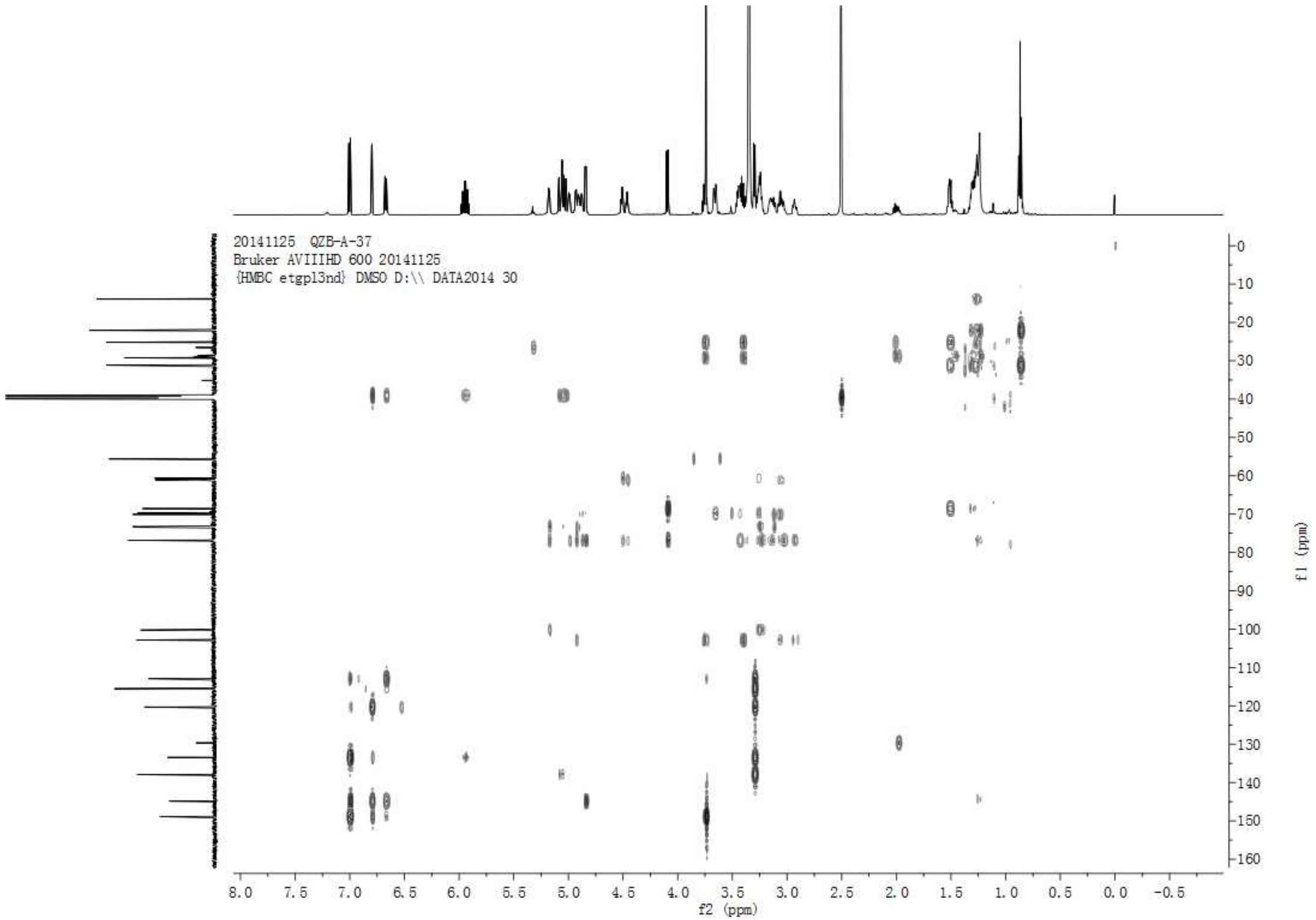

Figure S12. The HMBC Spectrum of Compound **2** in DMSO-d_6 (600 MHz for ^{1}H).

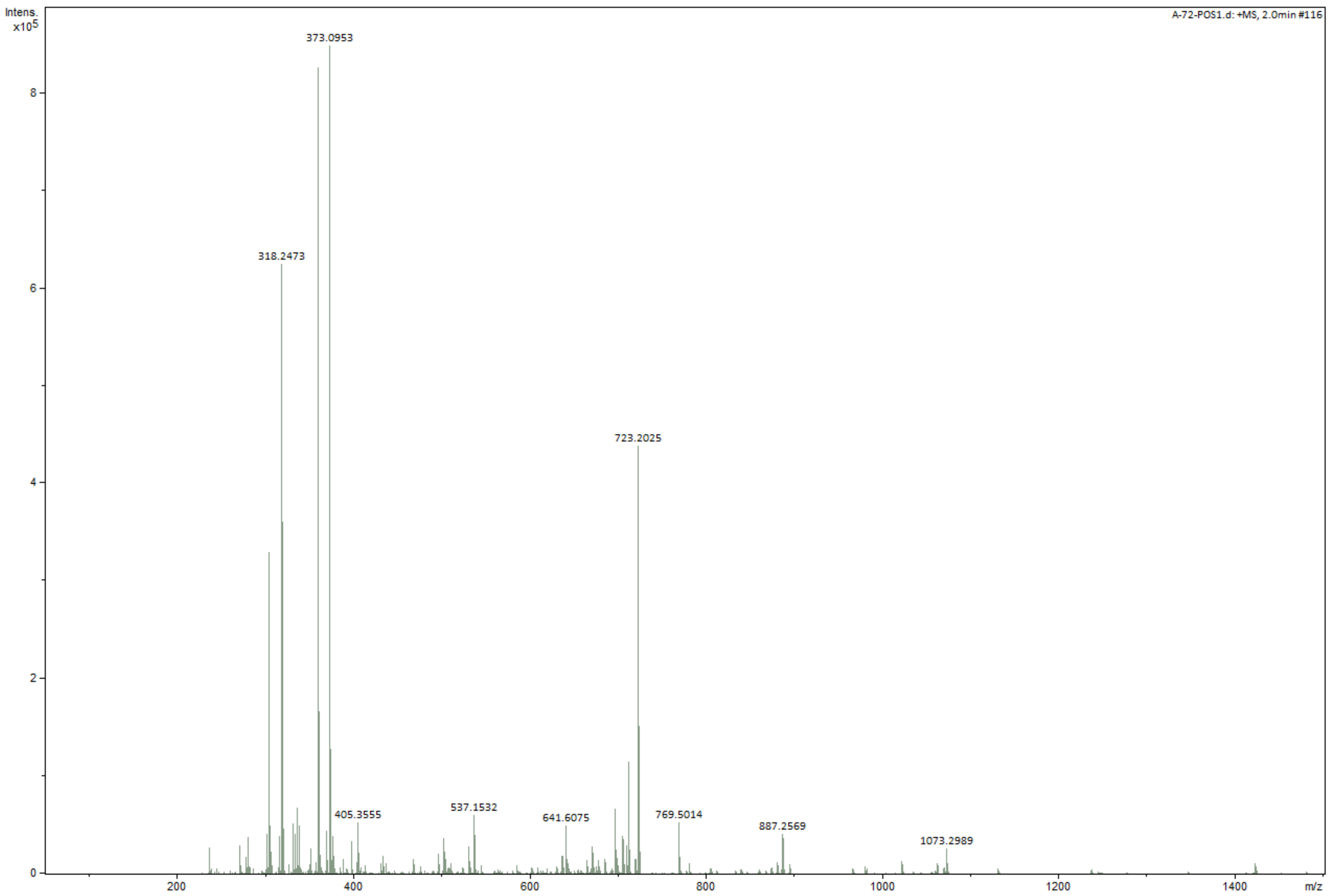

Figure S13. The HREIMS Spectroscopic Data of Compound **3.**

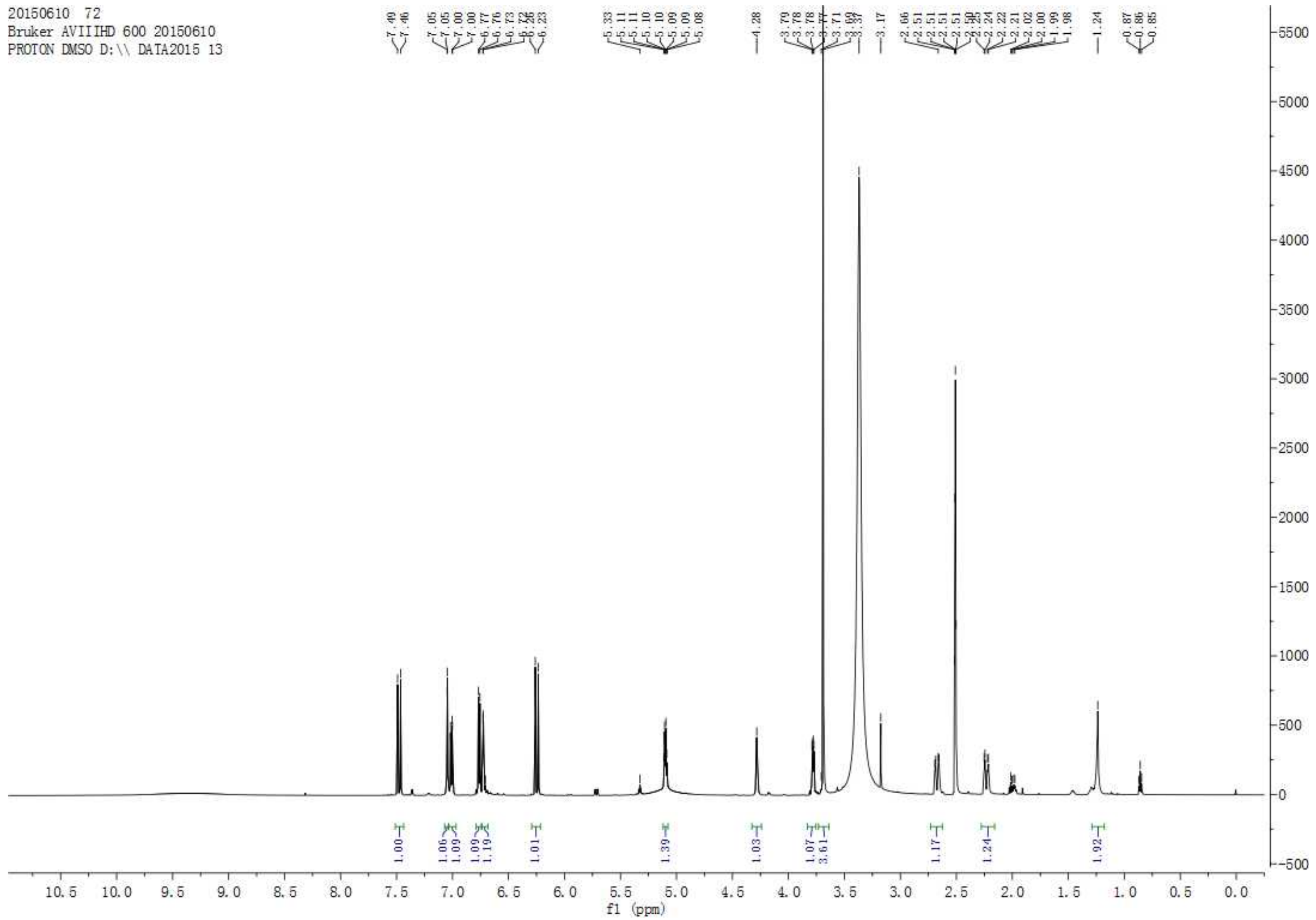

Figure S14. The ^{1}H NMR Spectrum of Compound **3** in DMSO-d_6 (600 MHz).

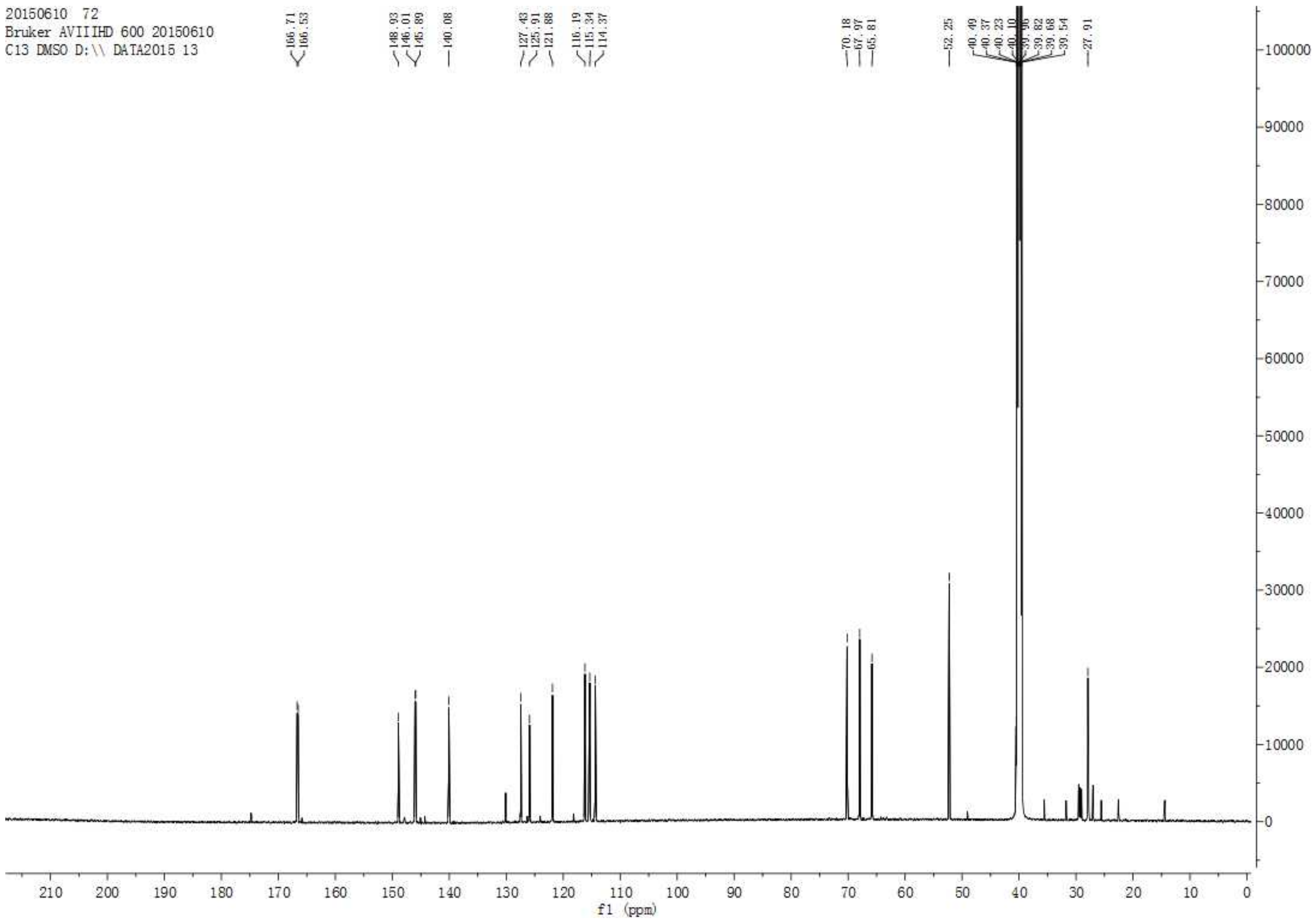

Figure S15. The ^{13}C NMR Spectrum of Compound **3** in DMSO-d_6 (150 MHz)

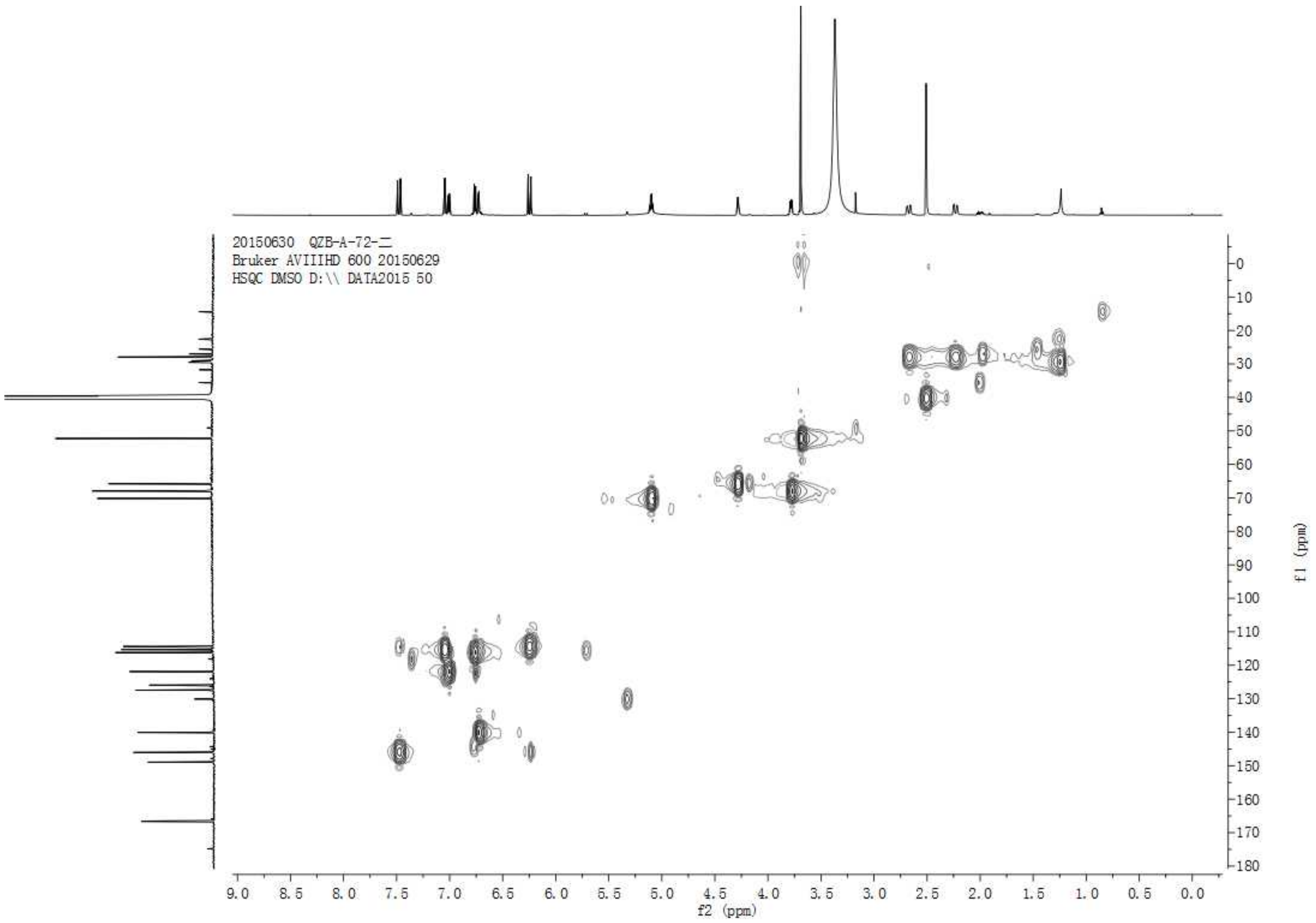

Figure S16. The HSQC Spectrum of Compound **3** in DMSO-d_6 (600 MHz for ^{1}H)

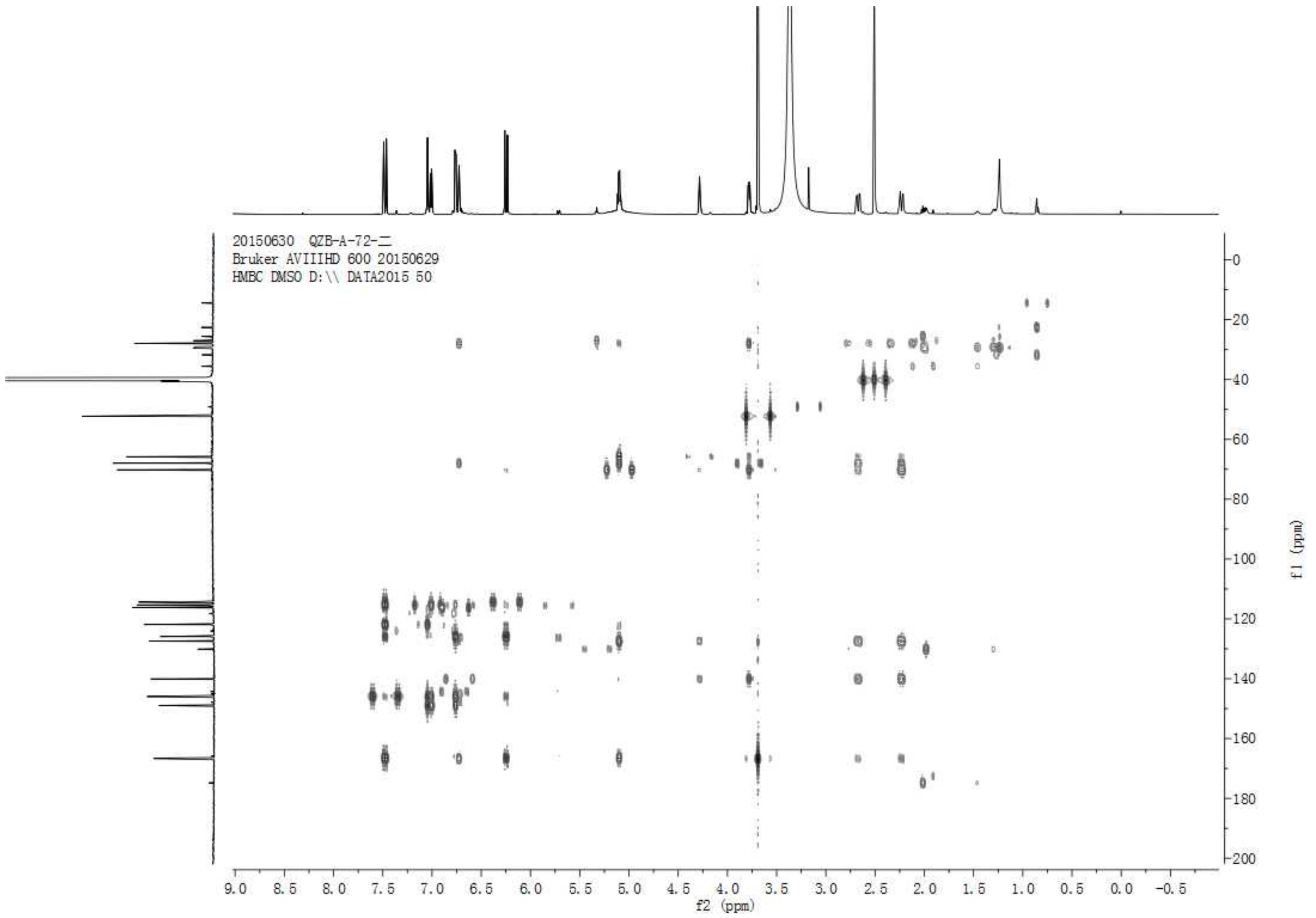

Figure S17. The HMBC Spectrum of Compound **3** in DMSO-d_6 (600 MHz for ^{1}H)

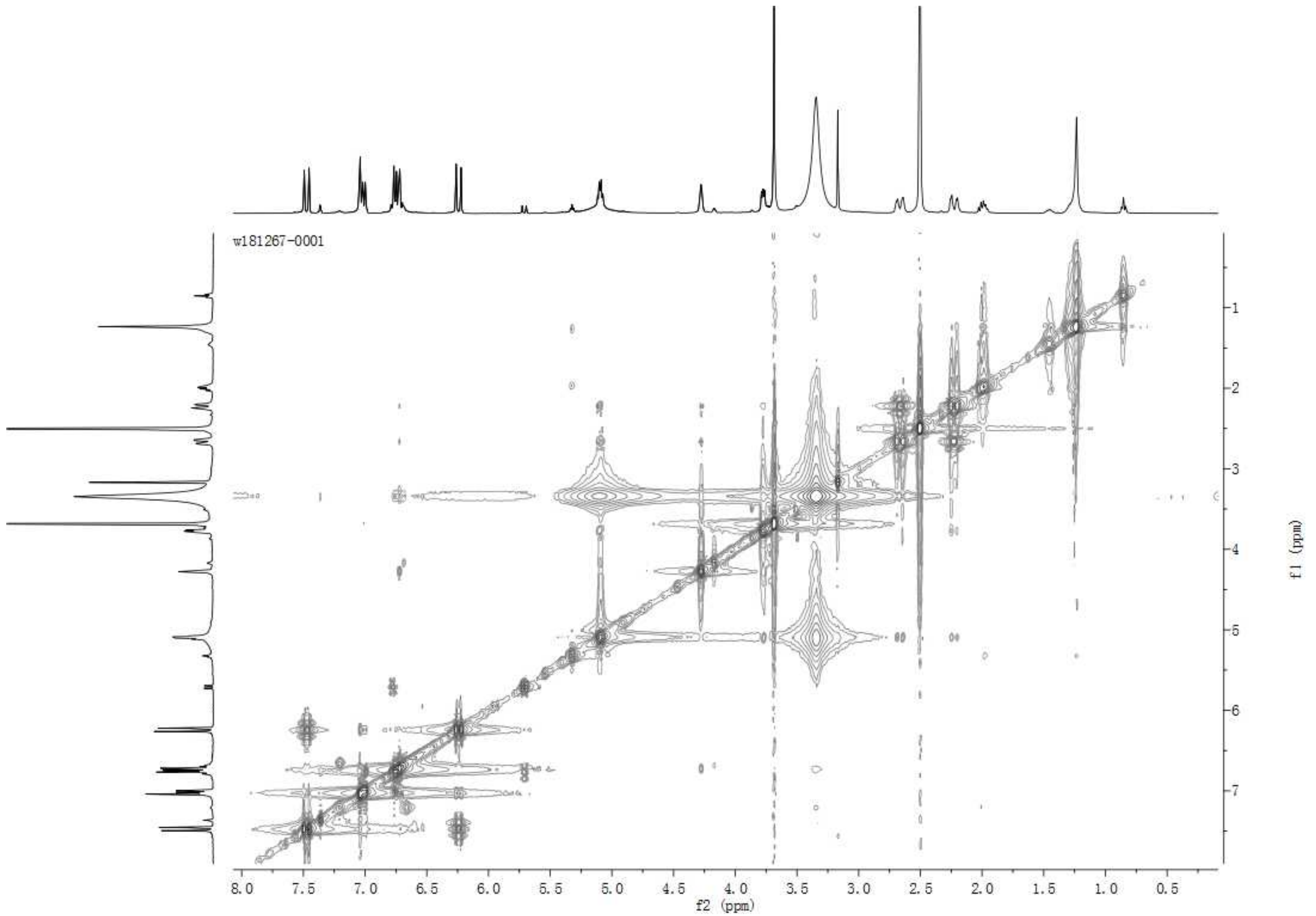

Figure S18. The NOESY Spectrum of Compound **3** in DMSO-d_6 (600 MHz)

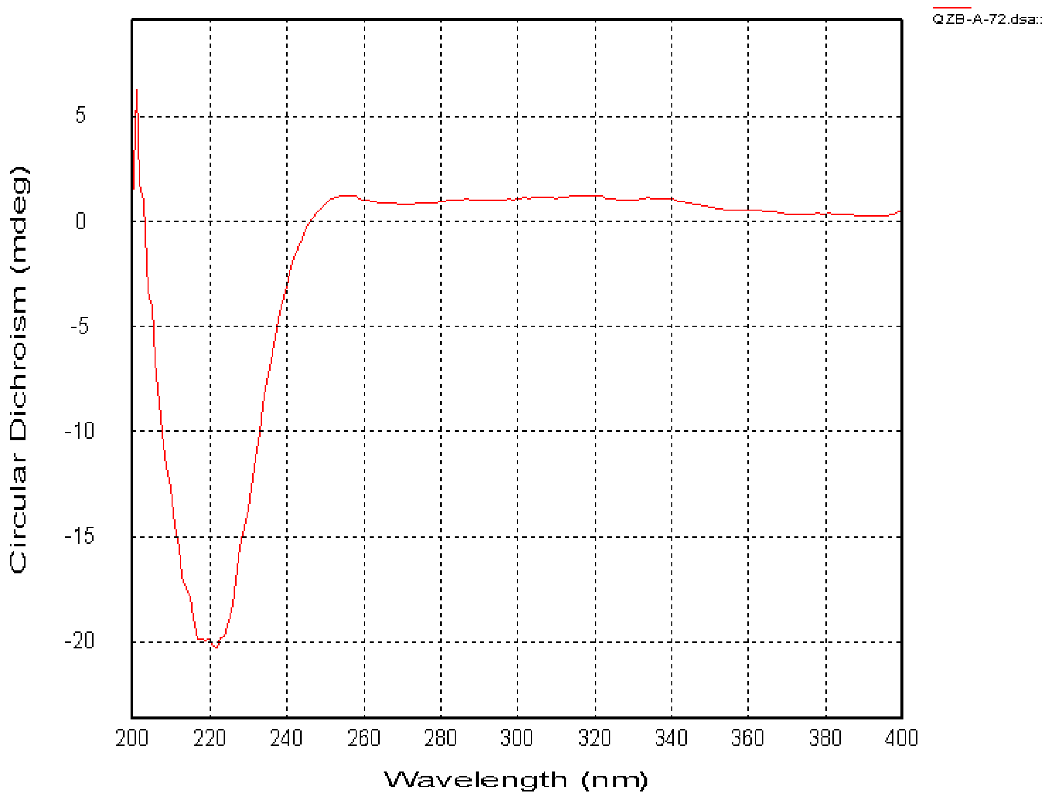

Figure S19. The ECD Spectrum of Compound **3** in MeOH
